# NuRD Suppresses Pluripotency Gene Expression to Promote Transcriptional Heterogeneity and Lineage Commitment

**DOI:** 10.1016/j.stem.2012.02.020

**Published:** 2012-05-04

**Authors:** Nicola Reynolds, Paulina Latos, Antony Hynes-Allen, Remco Loos, Donna Leaford, Aoife O'Shaughnessy, Olukunbi Mosaku, Jason Signolet, Philip Brennecke, Tüzer Kalkan, Ita Costello, Peter Humphreys, William Mansfield, Kentaro Nakagawa, John Strouboulis, Axel Behrens, Paul Bertone, Brian Hendrich

**Affiliations:** 1 Wellcome Trust Centre for Stem Cell Research, University of Cambridge, Tennis Court Road, Cambridge CB2 1QR, UK; 2 Stem Cell Institute, University of Cambridge, Tennis Court Road, Cambridge CB2 1QR, UK; 3 Department of Biochemistry, University of Cambridge, Tennis Court Road, Cambridge CB2 1QR, UK; 4 EMBL European Bioinformatics Institute, Wellcome Trust Genome Campus, Cambridge CB10 1SD, UK; 5 Institute for Stem Cell Research and MRC Centre for Regenerative Medicine, University of Edinburgh, Edinburgh EH9 3JQ, UK; 6 London Research Institute, Lincoln's Inn Fields Laboratories, London WC2A 3PX, UK; 7 Institute of Molecular Oncology, BSRC “Alexander Fleming,” P.O. Box 74145, Varkiza, Greece; 8 Genome Biology and Developmental Biology Units, European Molecular Biology Laboratory, Meyerhofstrasse 1, 69117 Heidelberg, Germany

## Abstract

Transcriptional heterogeneity within embryonic stem cell (ESC) populations has been suggested as a mechanism by which a seemingly homogeneous cell population can initiate differentiation into an array of different cell types. Chromatin remodeling proteins have been shown to control transcriptional variability in yeast and to be important for mammalian ESC lineage commitment. Here we show that the Nucleosome Remodeling and Deacetylation (NuRD) complex, which is required for ESC lineage commitment, modulates both transcriptional heterogeneity and the dynamic range of a set of pluripotency genes in ESCs. In self-renewing conditions, the influence of NuRD at these genes is balanced by the opposing action of self-renewal factors. Upon loss of self-renewal factors, the action of NuRD is sufficient to silence transcription of these pluripotency genes, allowing cells to exit self-renewal. We propose that modulation of transcription levels by NuRD is key to maintaining the differentiation responsiveness of pluripotent cells.

## Graphical abstract

**Graphical Abstract:**

**Figure:**
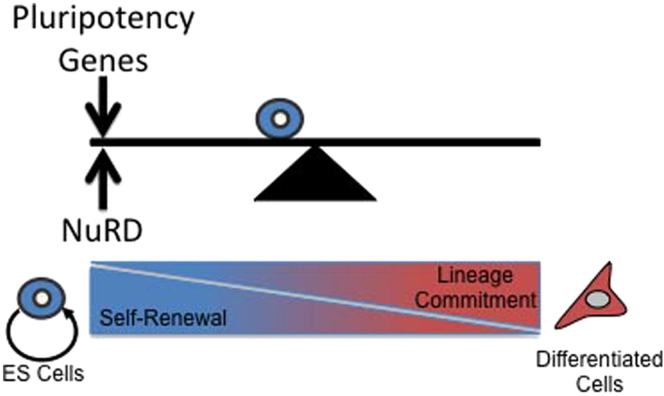

**Highlights:**

► NuRD directly regulates the transcription of pluripotency genes ► The repressive activity of NuRD is required for ESC differentiation ► NuRD mediates transcriptional heterogeneity in ESC populations ► Transcription levels are determined by balanced activation and silencing

## Introduction

Embryonic stem cells (ESCs) have the ability to differentiate into any cell type in an adult animal, a trait known as pluripotency. They are also able to self-renew, or to proliferate indefinitely in culture without losing their developmental potential. There is considerable hope that human ESCs and induced pluripotent stem cells (iPSCs) will provide both a model system for better understanding early human development and a source of human tissue to be used in drug screening and for studying disease progression (Yamanaka, 2009). In order to realize the therapeutic potential of ESCs and iPSCs, it will be essential to be able to control both their exit from the self-renewal program and their subsequent commitments to particular developmental lineages. Entry into lineage-specific differentiation from the pluripotent state is pivotal to mammalian development, yet the molecular mechanisms behind control of lineage commitment remain poorly understood.

ESCs grown in standard conditions (i.e., in growth medium supplemented with bovine serum and the cytokine Leukemia Inhibitory Factor, or LIF) contain subpopulations of differentiating cells, despite the fact that the majority of cells in the culture are undergoing self-renewal. Thus, functional heterogeneity exists in a culture of genetically identical cells being exposed to uniform culture conditions, indicating that stochastic events may be involved in exiting self-renewal. Recently it has emerged that ESCs grown in serum and LIF conditions express variable levels of a number of pluripotency-associated transcription factors, and that the expression levels of some of these genes correlate with the differentiation potential of a cell (Chambers et al., 2007; Hayashi et al., 2008; Toyooka et al., 2008). Notably, ESCs grown in fully defined media containing inhibitors of the fibroblast growth factor (FGF)/mitogen-activated protein kinase (Mek)/extracellular signal-related kinase (Erk1/2; Mapk3/1) pathway and glycogen synthase kinase 3 (Gsk3) (2i media; Nichols et al., 2009; Ying et al., 2008) display neither a propensity for differentiation nor transcriptional heterogeneity of pluripotency factors (Wray et al., 2010). Thus transcriptional heterogeneity within cell populations is tightly linked to cellular heterogeneity, and the former could underlie the emergence of the latter.

The differences between lineage-committed cells and the pluripotent cells from which they originated are largely defined by gene expression patterns. Evidence for this comes from the demonstration that pluripotency can be induced in somatic cells via introduction of only four transcription factors (Takahashi and Yamanaka, 2006). Further evidence is provided by studies showing that pluripotent embryonic germ cell lines can be efficiently derived from embryonic gonads, which consist entirely of lineage-committed cells (Leitch et al., 2010; Matsui et al., 1992; Resnick et al., 1992). It therefore stands to reason that proteins involved in the control of transcription will play important roles in lineage commitment and differentiation of pluripotent cells. Consistent with this hypothesis, several proteins involved in transcriptional regulation have been shown to be important for early embryonic viability and for ESC lineage commitment or pluripotency (reviewed in McDonel et al., 2009; Niwa, 2007).

The Nucleosome Remodeling and Deacetylation (NuRD) corepressor complex is an example of a transcriptional modulator that has been shown to be required for developmental transitions of pluripotent cells in both peri-implantation stage embryos and ESCs (Kaji et al., 2006, 2007; McDonel et al., 2009). NuRD-mediated silencing is predominantly associated with developmental decisions in a variety of different contexts. In species such as flies and worms, NuRD components have been shown to play important roles in signaling pathways and in tissue patterning (Ahringer, 2000; McDonel et al., 2009). In mice, NuRD-mediated silencing has been implicated in cell fate decisions in somatic stem cells (Kashiwagi et al., 2007; Yoshida et al., 2008) and in developmental transitions during peri-implantation development (Kaji et al., 2007).

The scaffold protein Mbd3 is essential for proper assembly of the NuRD complex (Kaji et al., 2006; Zhang et al., 1999). *Mbd3^−/−^* ESCs are viable and maintain expression of genes associated with pluripotent cells, but continue to self-renew when induced to differentiate via removal of self-renewal factors (Kaji et al., 2006). NuRD has been shown to maintain the barrier between embryonic and trophoblast cell fates in ESCs (Latos et al., 2012; Zhu et al., 2009). Importantly, while cells lacking NuRD activity do gain the ability to form trophoblast cells, in the absence of trophoblast-inducing external stimuli, they remain as self-renewing ESCs (Latos et al., 2012).

In this study we address the question of how NuRD-mediated transcriptional regulation facilitates lineage commitment of ESCs. We find that NuRD directly regulates the expression levels of a number of pluripotency genes in ESCs. Rather than completely silencing these targets, however, we provide evidence that NuRD is required to attenuate transcript levels below a threshold that allows exit from pluripotency, thus sensitizing cells to a loss of self-renewal factors. We further show that it is the interplay between variable transcriptional activation signals and the repressive influence of the NuRD complex that results in transcriptional heterogeneity at pluripotency-associated genes in ESC cultures.

## Results

### NuRD Directly Regulates Expression of Pluripotency Genes in ESCs

**Figure 1:**
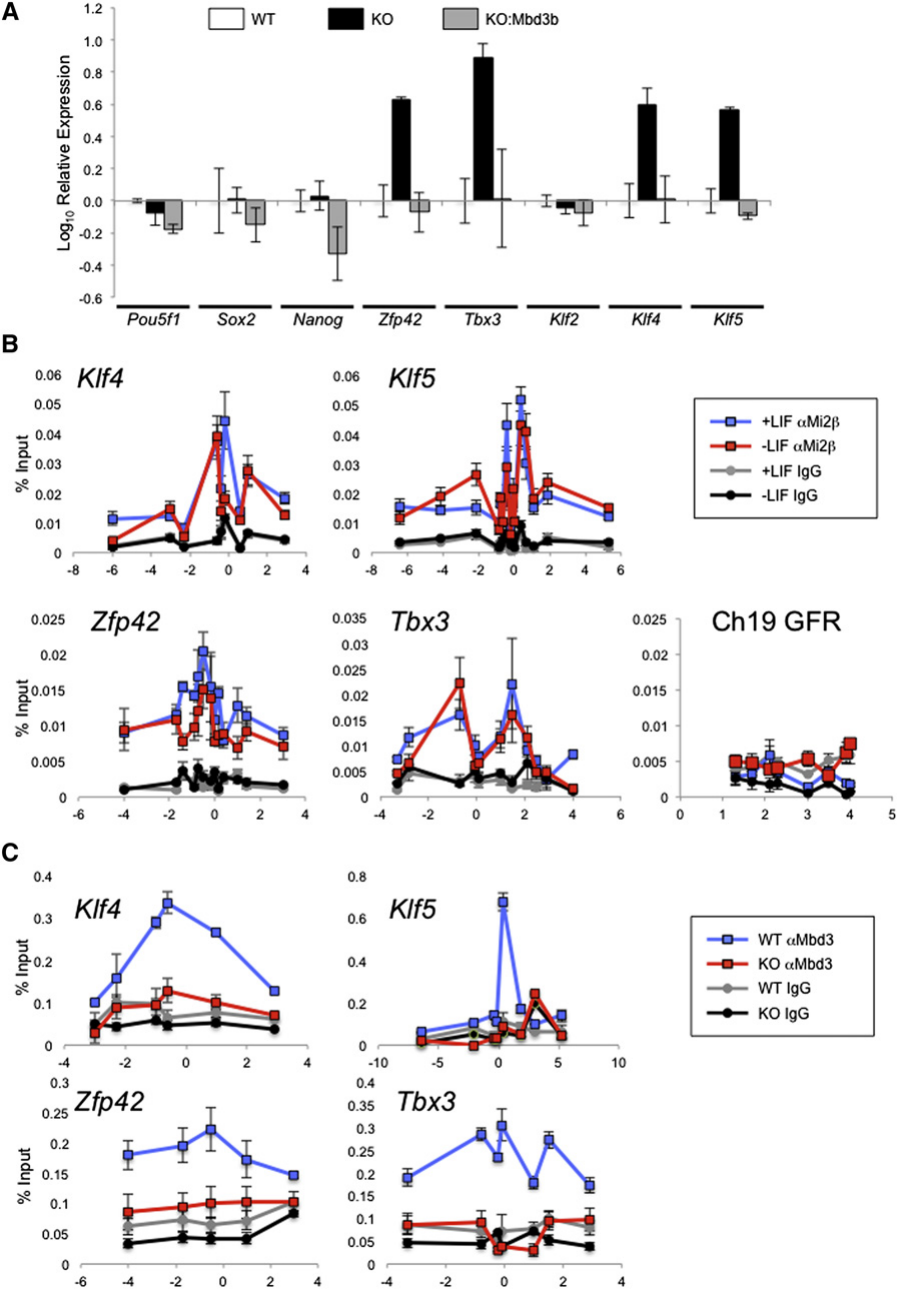
NuRD Controls Expression Levels of Pluripotency Genes in ESCs (A) Expression of indicated genes in wild-type ESCs (WT), *Mbd3^−/−^* ESCs (KO), and *Mbd3^−/−^* ESCs rescued with an *Mbd3b* transgene (KO:Mbd3b), relative to the expression levels in wild-type ESCs grown in LIF and serum conditions. (B) Chromatin immunoprecipitation (ChIP) was performed using anti-Mi2β or a mouse IgG control antibody in wild-type ESCs grown in self-renewing conditions (+LIF) or after 24 hr of LIF withdrawal (−LIF). Immunoprecipitates were probed with primer pairs located across the indicated gene promoters and into the body of the genes and plotted as percentage of input (y axis). Numbers along the x axis indicate distance relative to transcription start site for indicated genes. “Ch19 GFR” refers to a gene-free region on chromosome 19 (Nóbrega et al., 2004). (C) ChIP using anti-Mbd3 or mouse IgG control antibody in wild-type (WT) or *Mbd3^−/−^* (KO) ESCs grown in standard serum and LIF conditions. See also Figure S1.

Overexpression of a number of genes in ESCs has previously been shown to reduce or remove a dependency of ESCs upon LIF for self-renewal (Chambers et al., 2003; Ema et al., 2008; Hall et al., 2009; Li et al., 2005; Niwa et al., 2009; Zhang et al., 2008). Similarly, ESCs lacking the structural NuRD component protein Mbd3 are capable of LIF-independent self-renewal (Kaji et al., 2006). We therefore hypothesized that NuRD activity is required to restrict expression of pluripotency-associated genes. Although mRNA levels of the canonical pluripotency genes *Pou5f1*, *Nanog*, and *Sox2* were not significantly increased in *Mbd3^−/−^* compared to those in wild-type ESCs, we found increases in the expression levels of *Zfp42* (*Rex1*), *Tbx3*, *Klf4*, and *Klf5* in Mbd3 mutant ESCs (Figure 1A). In all cases expression of these genes was reduced to normal or lower levels when an *Mbd3* transgene was introduced into the mutant cells (“rescued“ ESCs; Kaji et al., 2006), demonstrating that the observed expression changes correlate with the presence or absence of NuRD function.

To determine whether this control of active transcription is exerted directly or indirectly by the NuRD complex, we assessed NuRD component binding to the promoters of misexpressed genes in ESCs using chromatin immunoprecipitation (ChIP). The presence of the NuRD components Mi2β and Mbd3 was detected at the promoters and gene bodies of pluripotency-associated genes in undifferentiated ESCs (Figures 1B and 1C; Figure S1 available online). While both Mi2β and Mbd3 binding was most apparent near transcription start sites, association of both proteins could be detected in a broad region encompassing the promoters and gene bodies of these targets, consistent with previous studies in both mouse and insect cells (Miccio et al., 2010; Murawska et al., 2011; Reynolds et al., 2012). Mi2β association remained 24 hr after LIF withdrawal from standard media at all genes tested (Figure 1B), indicating that NuRD activity serves both to attenuate expression of these genes during self-renewal and to reinforce downregulation as cells lose positive regulators and commit to differentiate.

**Figure 2:**
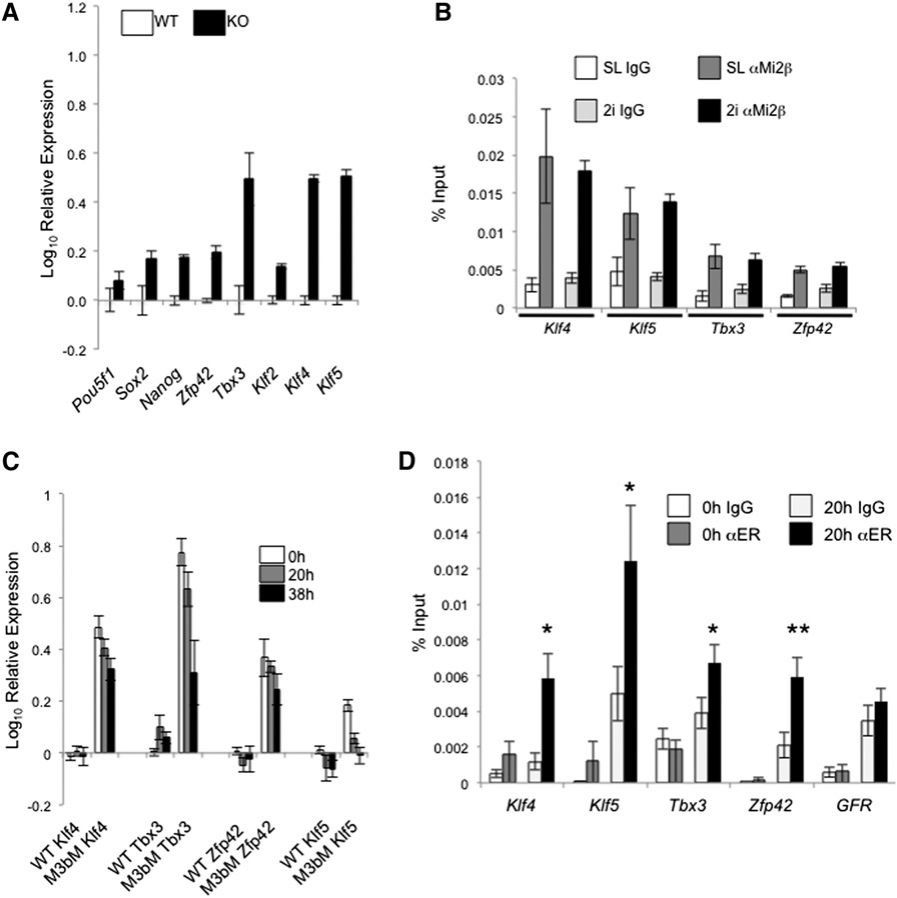
NuRD Restricts Expression Levels of Actively Transcribed Genes (A) Expression of indicated genes in *Mbd3^−/−^* ESCs grown in 2i/LIF relative to wild-type cells grown in the same conditions. (B) ChIP was performed using anti-Mi2β or a mouse IgG control antibody in wild-type ESCs grown in serum and LIF (SL) or in 2i/LIF conditions and probed with primers located at the transcription start sites for the indicated genes. (C) Expression of indicated genes in wild-type (WT) or *Mbd3^−/−^* ESCs expressing MER-Mbd3b-MER (M3bM) prior to tamoxifen treatment (0 hr) and after 20 or 38 hr of tamoxifen treatment, relative to the expression levels in wild-type ESCs. (D) MER-Mbd3b-MER goes to NuRD target genes after tamoxifen addition. ChIP was performed using anti-ER or a mouse IgG control antibody in *Mbd3^−/−^* ESCs expressing MER-Mbd3b-MER either in the absence of tamoxifen (0 hr) or after 20 hr of tamoxifen treatment (20 hr), which were then probed with primers located at the transcription start sites for the indicated genes. “GFR” refers to the chromosome 19 gene free region. ^∗^p < 0.05, ^∗∗^p < 0.005. Error bars represent standard error of the mean (SEM).

NuRD is known as a transcriptional silencer; however, our expression data indicate that NuRD acts to restrict expression levels of a set of actively transcribed genes, rather than repress them completely. *Zfp42*, *Tbx3*, and *Klf4* all show heterogeneous expression patterns in ESCs grown in serum and LIF conditions (Toyooka et al., 2008). Changes in overall expression levels in *Mbd3^−/−^* ESCs could therefore be due either to the loss of silencing in the subpopulation of cells normally exhibiting low expression levels, or to the modulation of active transcription across the population as a whole. To distinguish between these possibilities we assessed the relative expression levels of these same genes in wild-type and mutant ESCs grown in 2i media supplemented with LIF (2i/LIF). ESCs maintained in 2i/LIF conditions do not spontaneously differentiate and display a uniformly high level of Nanog and Rex1 expression (Wray et al., 2010). As shown in Figure 2A, all pluripotency markers tested showed an increase in transcript levels in *Mbd3^−/−^* ESCs relative to wild-type cells, with *Tbx3*, *Klf4*, and *Klf5* again exhibiting the most pronounced effects. Mi2β association was also found at these genes by ChIP in ESCs grown in 2i/LIF conditions (Figure 2B). Since cells grown in 2i/LIF completely lack the low-expressing subpopulation, these data confirm that NuRD indeed functions to restrict active transcription of these pluripotency-associated genes in ESCs.

It is possible that ESCs maintained in culture for long periods would undergo adaptive changes in response to the loss of NuRD activity. To verify that expression of pluripotency-associated genes is acutely responsive to the presence or absence of NuRD function, we took advantage of *Mbd3^−/−^* ESCs expressing an inducible Mbd3 protein (isoform b, Mbd3b) (Reynolds et al., 2012). In these cells exogenous Mbd3b is fused at both its N and C termini to the mouse estrogen receptor (MER), resulting in the protein being sequestered in the cytoplasm. Upon addition of 4-hydroxytamoxifen, MER-Mbd3b-MER translocates into the nucleus, restoring NuRD function and NuRD-mediated gene silencing (Reynolds et al., 2012). Using this system we observed recruitment of NuRD to the promoter regions, together with a reduction in expression levels, of *Klf4*, *Klf5*, *Tbx3*, and *Zfp42* within 20 hr of 4-hydroxytamoxifen exposure (Figures 2C and 2D). We therefore conclude that NuRD-mediated control of pluripotency gene expression is not an artifact of long-term culture.

### Elevated Expression of Pluripotency Genes Prevents Lineage Commitment of *Mbd3^−/−^* ESCs

**Figure 3:**
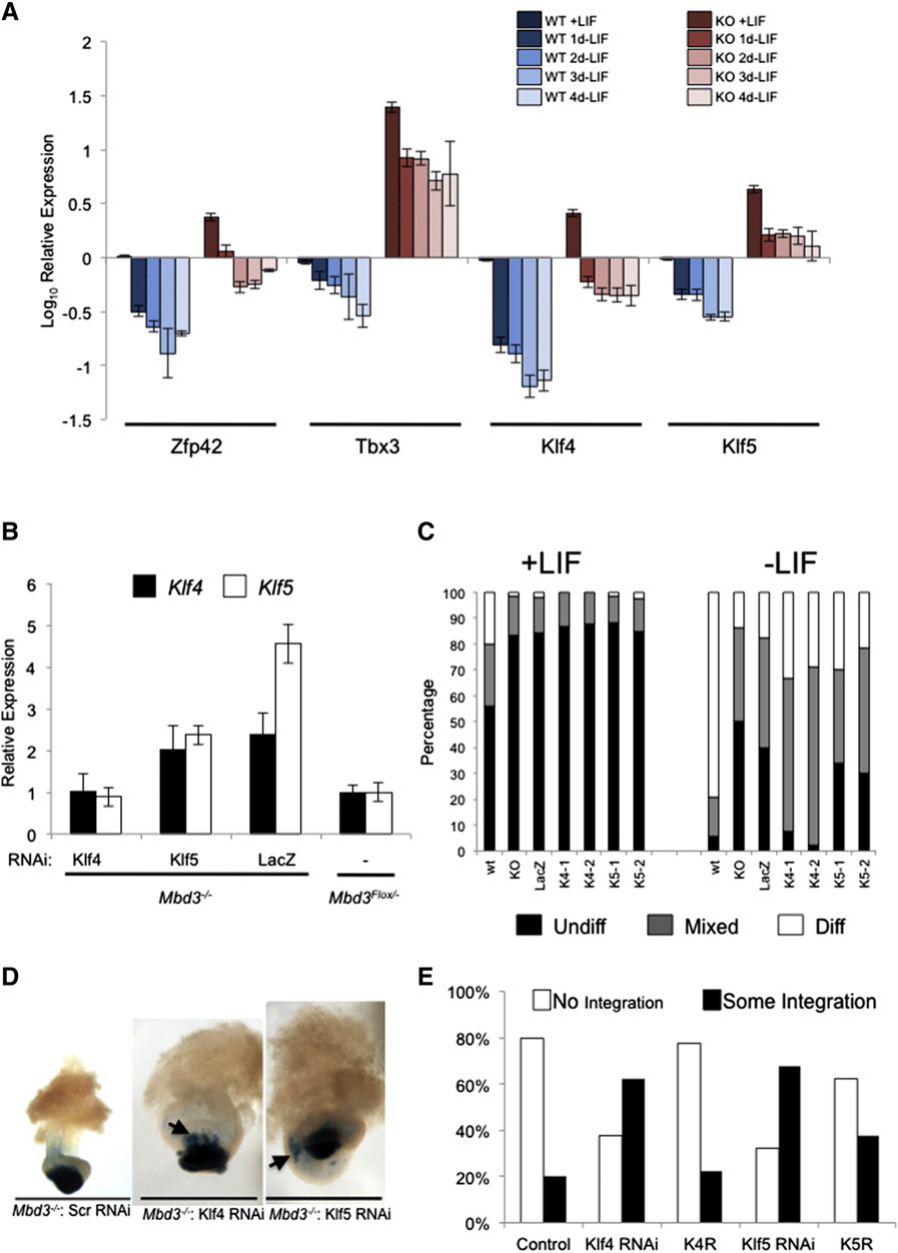
Misexpression of NuRD Target Genes Contributes to the Differentiation Defect of *Mbd3^−/−^* ESCs (A) Expression of indicated genes in wild-type and *Mbd3^−/−^* ESCs in serum and LIF or in the absence of LIF for the indicated times is plotted relative to expression in wild-type cells prior to LIF withdrawal. Error bars represent SEM from ≥3 experiments performed on different wild-type and mutant ESC lines. See also Figure S2. (B) Expression levels of *Klf4* (black bars) and *Klf5* (white bars) in *Mbd3* heterozygous ESCs (Flox/−) or *Mbd3^−/−^* ESC lines expressing microRNAs directed against *Klf4*, *Klf5*, and *LacZ* are displayed relative to expression levels seen in *Mbd3^Flox/−^* ESCs. Error bars represent SEM. (C) An alkaline phosphatase (AP) assay was performed using *Mbd3^Flox/−^* ESCs (referred to as WT for simplicity), *Mbd3^−/−^* ESCs (KO), and two different *Mbd3^−/−^* ESC lines each expressing microRNAs directed against *Klf4* (K4-1 or K4-2) or *Klf5* (K5-1 or K5-2), as well as one cell line expressing microRNAs against LacZ. The proportions of fully undifferentiated colonies staining uniformly for AP are represented in black (Undiff), partially differentiated colonies showing heterogeneous AP staining are in gray (Mixed), and fully differentiated colonies are in white (Diff). (D) Chimeric embryos made by aggregating indicated ESC lines with wild-type embryos dissected at 8.5 dpc. The presence of ESC-derived tissue is indicated by LacZ staining (blue). Areas where ES-derived cells have integrated with host embryos are indicated with arrows. Scale bars represent 1 mm. Images are representative of multiple examples of chimeric embryos. (E) Quantitation of chimera experiments. The percentage of chimeric embryos displaying little or no integration (No Integration; white columns) of ESCs with the host cells or significant integration (Some Integration; black columns) of ESCs with the host embryo are plotted for *Mbd3^−/−^* ESCs expressing a scrambled siRNA (“Control,” n = 25), siRNA directed against Klf4 (“Klf4 RNAi,” n = 24) or Klf5 (“Klf5 RNAi,” n = 28), Klf4 RNAi cells rescued by expression of an RNAi-resistant Klf4 cDNA (“K4R,” n = 9), and Klf5 RNAi cells rescued by expression of an RNA-resistant Klf5 cDNA (“K5R,” n = 24). Scoring for contribution was performed blind to the ESC genotype.

If the misexpression of these pluripotency genes contributes toward the LIF-independent self-renewal phenotype displayed by *Mbd3^−/−^* ESCs, then overexpression should persist in conditions that would normally induce lineage commitment. Because expression of *Zfp42, Tbx3*, *Klf4*, and *Klf5* has been shown to be stimulated by LIF signaling (Hall et al., 2009; Niwa et al., 2009; Toyooka et al., 2008), we monitored expression levels of these genes in *Mbd3^−/−^* ESCs over a time course of LIF withdrawal. LIF withdrawal for 24 hr was sufficient to attenuate transcriptional activation mediated by Stat3, the downstream effector of the LIF signaling pathway (Niwa et al., 1998), in both wild-type and *Mbd3^−/−^* cells. This was verified both by a decrease in expression of the Stat3 target gene *Socs3* and by loss of Stat3 binding to the *Socs3* promoter (Figure S2), demonstrating that *Mbd3^−/−^* ESCs remain sensitive to the presence or absence of LIF stimulation. Like wild-type ESCs, *Mbd3^−/−^* ESCs displayed an abrupt downregulation of *Zfp42, Tbx3*, *Klf4*, and *Klf5* 24 hr after LIF withdrawal (Figure 3A), indicating that these genes remain sensitive to Stat3-mediated activation in the absence of functional NuRD complex. Nevertheless, expression of all four genes remained elevated in *Mbd3^−/−^* cells compared to wild-type cells, and little or no ongoing decrease was seen beyond the initial drop upon LIF withdrawal. However, while *Zfp42, Tbx3*, *Klf4*, and *Klf5* remain sensitive to LIF-mediated transcriptional activation in *Mbd3^−/−^* ESCs, complete silencing of the genes upon loss of the transcriptional activation signal does not occur, demonstrating a requirement for functional NuRD in this process.

To determine the biological relevance of this failure to completely silence pluripotency gene expression, we assessed the ability of *Mbd3^−/−^* ESCs to differentiate when the transcript levels of *Klf4* or *Klf5* were reduced to approximately wild-type levels (Figure 3B). *Mbd3^−/−^* ESCs and those expressing an RNAi construct directed against an irrelevant transcript (encoding LacZ) produced 40%–50% undifferentiated colonies when plated at clonal density in the absence of LIF for 4 days, whereas under the same conditions, wild-type cells produced almost exclusively differentiated colonies (Figure 3C). Knocking down *Klf4* expression in *Mbd3^−/−^* cells, however, resulted in a marked rescue of the differentiation defect, in that nearly all colonies contained differentiated cells (Figure 3C). In contrast, knocking down *Klf5* in *Mbd3^−/−^* ESCs had no rescuing effect in this assay. Notably, knockdown of *Klf4* in *Mbd3^−/−^* ESCs also resulted in reduction of *Klf5* transcript levels, whereas knockdown of *Klf5* had no effect on *Klf4* transcript levels (Figure 3B).

In addition to displaying persistent self-renewal upon removal of LIF in culture, *Mbd3^−/−^* ESCs fail to contribute toward embryonic development in chimeric embryos (Kaji et al., 2006). When aggregated with a wild-type morula, *Mbd3^−/−^* ESCs fail to mix with host cells, prevent host cells from forming an embryo, and on their own form only a very rudimentary primitive ectoderm-like structure (Figure 3D). However, *Mbd3^−/−^* cells in which either *Klf4 or Klf5* transcript levels were reduced via RNAi showed an increased ability to integrate into host embryos as compared to controls (Figures 3D and 3E). This reduction in phenotype severity by *Klf4* or *Klf5* knockdown was reversed by overexpression of an RNAi-resistant cDNA in both the *Klf4* and *Klf5* knockdown cell lines (Figure 3E). This demonstrates that inappropriate expression levels of both *Klf4* and *Klf5* contribute to the differentiation defect apparent in *Mbd3^−/−^* ESCs. The resulting chimeric embryos are, nevertheless, severely abnormal, indicating that while knocking down these *Klf* genes results in partial rescue, aberrant transcription of other genes is also likely to play a part in the Mbd3 mutant phenotype. We conclude that NuRD-mediated control of *Klf4* and *Klf5* expression, in addition to that of other genes, facilitates lineage commitment of ESCs.

### NuRD Maintains Transcriptional Heterogeneity in ESC Populations

**Figure 4:**
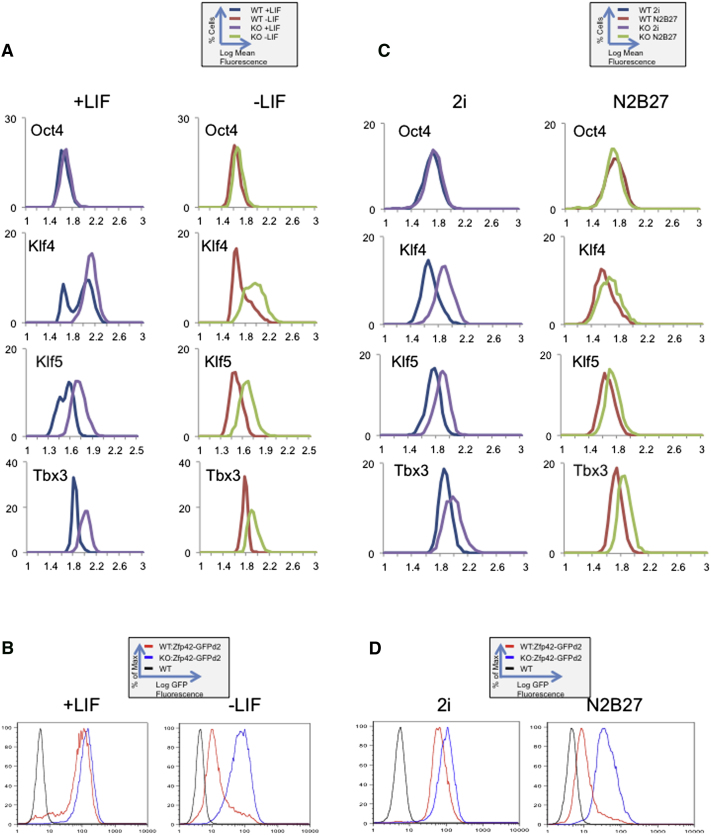
NuRD Controls Transcriptional Heterogeneity in ESCs (A) Expression levels for indicated proteins were measured in ESC cultures by antibody staining and immunofluorescence microscopy. Log of relative fluorescence is plotted along the x axis, with the proportion of cells indicated along the y axis. Data is shown for wild-type (WT) and *Mbd3^−/−^* (KO) ESCs grown in self-renewing conditions (+LIF, left-hand panels) or after 48 hr in the absence of LIF (−LIF, right-hand panels). n > 4,000 cells for each line. (B) Flow cytometry analysis showing expression profiles of Zfp42-GFPd2 in a wild-type (WT: ZFP42-GFPd2) or *Mbd3^−/−^* (KO: Zfp42-GFPd2) background grown in standard media with 10% serum either with or without LIF (+LIF or –LIF, respectively). (C) Expression levels for indicated proteins were measured as in (A) for wild-type (WT) and *Mbd3^−/−^* (KO) ESCs maintained in 2i/LIF (2i, left-hand panels) or in the absence of inhibitors and LIF for 24 hr (N2B27, right-hand panels). n > 1,800 cells for each line. (D) Flow cytometry analysis showing expression profiles of Zfp42-GFPd2 in a wild-type (WT: ZFP42-GFPd2) or *Mbd3^−/−^* (KO: Zfp42-GFPd2) background in 2i/LIF (2i) or in defined media without inhibitors and LIF (N2B27). See also Figure S3.

ESCs grown in standard serum and LIF conditions normally display heterogeneous expression of several of the genes we have shown to be subjected to NuRD-mediated transcriptional control. While cells grown in 2i/LIF conditions show no such heterogeneity in expression, they are nevertheless subject to NuRD-mediated restriction of transcription levels. It is therefore possible that the influence of NuRD differs between subpopulations of ESCs, and that this variation would be undetectable across the population as a whole. To understand how these processes are affected at the single-cell level, we used immunofluorescence to measure protein abundance in individual cells from wild-type and Mbd3 mutant ESC cultures (Figures S3A and S3B). Using this method, we can class wild-type cells grown in serum and LIF into high- or low-expressing populations based upon Klf4 staining intensity (Figure 4A, left-hand panels). Quantification of Klf5 staining levels similarly reveals two populations of cells in wild-type cultures, although the high- and low-expressing populations are less distinct than for Klf4 (Figure 4A). In contrast, ESCs assessed in the same way based on Oct4 staining appear as a single, relatively uniform population (Figure 4A), as do cells stained for NuRD component proteins or the unrelated nuclear protein Sin3a (Figure S3C).

In the absence of a functional NuRD complex, two major changes to the Klf4 expression level distribution are apparent: first, the subpopulation of Klf4-negative cells is absent (Figure 4A). Second, there is an increase in the degree of Klf4 expression, i.e., the mean fluorescence intensity produced by Klf4-expressing cells is increased compared to that produced in wild-type cultures (p < 1*e*^−4^). NuRD activity is therefore important not only for generating the Klf4-low population, but also for restricting maximum expression levels in the Klf4-high population. This same pattern can be seen for the Klf5 protein (Figure 4A), whereas there is negligible change in the distribution of Oct4 expression in *Mbd3^−/−^* ESCs. Although Tbx3 has been reported to exhibit variable expression (Niwa et al., 2009), Tbx3 protein was not detectable by immunofluorescence in wild-type cells, but instead appears as a broad peak in *Mbd3^−/−^* ESCs (Figure 4A). Given the very short half-lives reported for both Klf4 and Klf5 proteins (∼2 hr; Chen et al. 2005a; Chen et al. 2005b) and the effect of *Mbd3* deletion upon *Klf4* and *Klf5* transcript levels (Figure 1), these changes in protein distributions likely reflect corresponding changes in transcriptional activity at both genes.

Culturing wild-type cells for 48 hr in the absence of LIF results in resolution of the two Klf4- or Klf5-expressing populations seen in self-renewing conditions toward the protein-low or -absent populations (Figure 4A, right-hand panels), consistent with a model in which cells expressing low levels of pluripotency genes are primed for differentiation (Kalmar et al., 2009). While curves produced in *Mbd3^−/−^* ESCs also shift toward reduced protein expression after LIF withdrawal, this is not to the extent seen in wild-type cells, and a distinct population of low-expressing cells never becomes evident (Figure 4A). However, if *Mbd3^−/−^* ESCs are forced to differentiate via exposure to retinoic acid, expression of both Klf4 and Oct4 is abolished in the majority of cells after 24 hr (Figure S3D), confirming that abrupt changes in protein levels are detectable in mutant cells using this system.

Given that Klf4 and Klf5 are both short-lived proteins, measuring protein abundance is likely to give a good indication of transcriptional output. To visualize *Zfp42* expression levels, however, we took advantage of a destabilized GFP reporter system (Wray et al., 2011) in which the coding region from one *Zfp42* allele is replaced with a destabilized enhanced GFP (GFPd2). This enables us to measure output from the *Zfp42* gene by flow cytometry for GFP fluorescence. Flow sorting of ESCs grown in serum and LIF expressing GFPd2 from the *Zfp42* locus reveals a large peak of GFP-positive cells, as well as a subpopulation of GFP-negative cells (Figure 4B, left-hand panel). ESCs lacking functional NuRD complex are unable to produce the GFP-negative population, and express higher average levels of Zfp42-GFPd2 than do wild-type ESCs, mimicking what was seen when quantifying Klf4 and Klf5 protein abundance by immunofluorescence. Wild-type cells maintained in the absence of LIF for 48 hr largely silence Zfp42-GFPd2 expression, whereas *Mbd3^−/−^* ESCs remain GFP positive (Figure 4B, right-hand panel).

ESCs grown in 2i/LIF conditions are far more homogeneous than those in standard conditions, consisting of only the protein-high-expressing population of cells. Nevertheless, ESCs in 2i/LIF conditions continue to display NuRD-dependent restriction of transcript levels for some pluripotency genes (Figure 2A). When protein fluorescence is quantified as above, wild-type ESCs cultured in 2i/LIF do indeed show more uniform patterns of Klf4, Klf5, Tbx3, and Zfp42-GFPd2 expression than cells grown in serum and LIF (Figures 4C and 4D, left-hand panels). The distributions of all four proteins in *Mbd3^−/−^* ESCs grown in 2i/LIF are shifted to the right as compared to those in wild-type cells, providing further evidence that NuRD limits active gene expression in self-renewing ESCs. After 24 hr in the absence of inhibitors and LIF, conditions that are permissive for differentiation, the distributions obtained for all four proteins are largely unchanged in either wild-type or *Mbd3^−/−^* ESCs, but in all cases are shifted to the left, indicating a uniform reduction in protein abundance across the cell populations (Figures 4C and 4D, right-hand panels).

Taken together, analyses of gene expression at the single-cell level indicate that the increase in steady state mRNA levels detected by quantitative RT-PCR in *Mbd3^−/−^* ESCs (Figure 1A and Figure 2A) is due to the failure to restrain transcription levels of actively transcribed genes irrespective of culture conditions, as well as a failure of gene silencing in a subpopulation of cells grown in serum and LIF conditions.

### NuRD Is a General Regulator of Transcriptional Heterogeneity in ESCs

**Figure 5:**
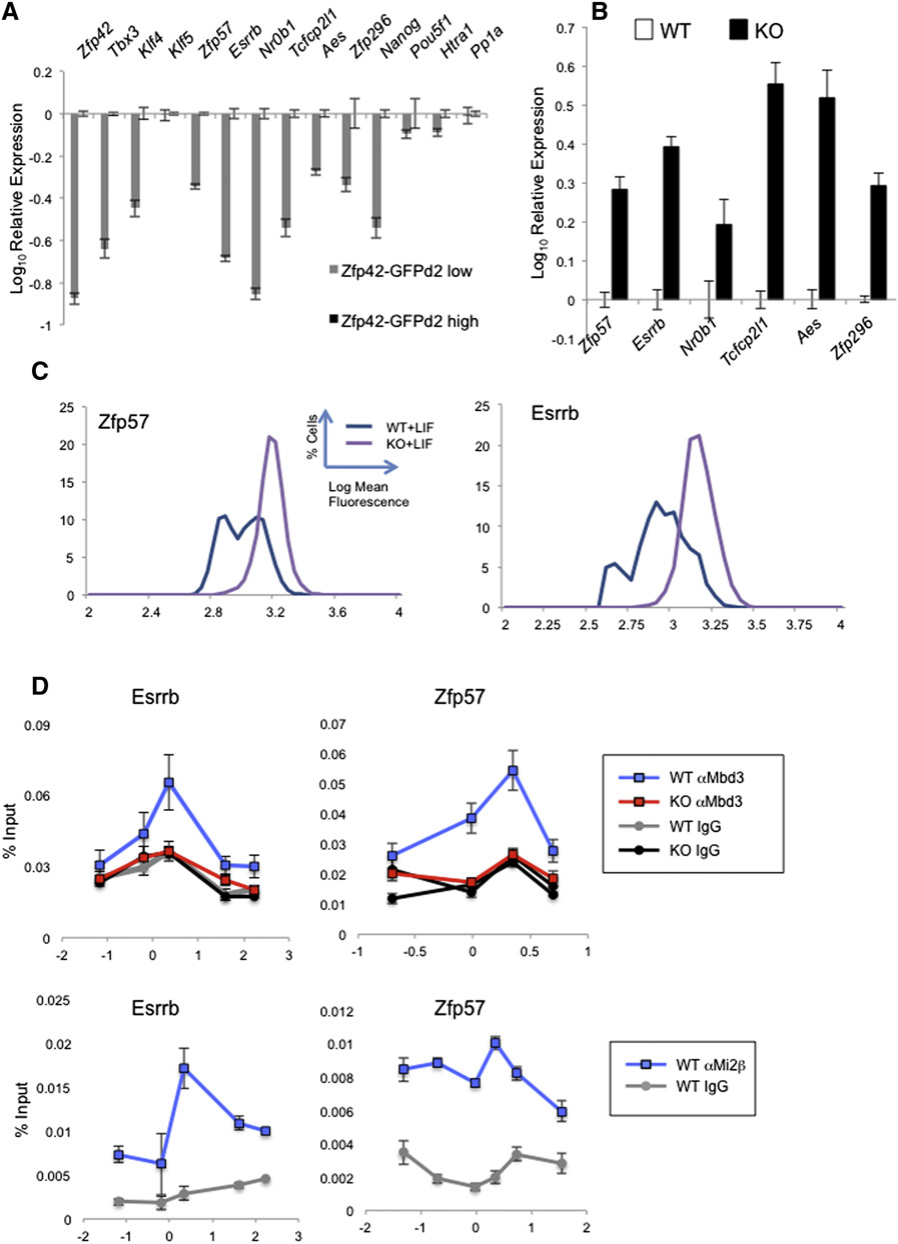
NuRD Is a General Regulator of Transcriptional Heterogeneity (A) Gene expression in Zfp42-GFPd2-low cells is expressed relative to expression in Zfp42-GFPd2-high cells (Marks et al., 2012). Included are genes identified by bioinformatic analysis (*Zfp42*, *Tbx3*, *Klf4*, *Zfp57*, *Esrrb*, *Nr0b1*, *Tcfcp2l1*, *Aes*, and *Zfp296*) as well as control pluripotency-associated genes (*Klf5*, *Nanog*, and *Pou5f1*) and one gene shown to be subject to NuRD-dependent transcriptional silencing in ESCs but not display transcriptional heterogeneity (*Htra1*; Reynolds et al., 2012). The latter two sets of genes do not display transcriptional heterogeneity in this assay. *Ppia* is a control housekeeping gene. (B) Expression of indicated genes in *Mbd3^−/−^* ESCs expressed relative to levels in wild-type ESCs. (C) Expression analysis for Zfp57 and Esrrb in wild-type (WT) and *Mbd3^−/−^* (KO) ESCs in serum and LIF conditions as in Figure 3A above. (D) ChIP was performed with anti-Mbd3 (top panels) and anti-Mi2β antibodies (bottom panels) as well as control IgG antibodies across the transcription start sites of *Esrrb* (left panels) and *Zfp57* (right panels) in wild-type (WT) or *Mbd3^−/−^* (KO; anti-Mbd3 ChIP only) ESCs grown in serum and LIF conditions. Immunoprecipitates were probed with primer pairs located across the indicated gene promoters and plotted as percentage of input (y axis). Numbers along the x axis indicate distance relative to major ES transcription start site for indicated genes in ESCs. See also Table S1.

Transcriptional heterogeneity has been demonstrated for relatively few genes in ESC populations, yet we have shown that NuRD modulates the expression patterns of at least four of these genes. To determine the extent to which transcriptional heterogeneity is regulated by NuRD activity, we compared mRNA-seq data from cells sorted for either high or low Zfp42 expression levels (Marks et al., 2012), and cross-referenced with results of mRNA sequencing from wild-type and *Mbd3^−/−^* ESCs. We identified 221 genes that were expressed at least 3-fold more greatly in Zfp42-GFPd2-high ESCs than in Zfp42-GFPd2-low ESCs and that also show a significant degree of misregulation in *Mbd3^−/−^* ESCs (Table S1). Changes in expression were confirmed by qRT-PCR for a number of these genes (Figures 5A and 5B). This set of genes is highly enriched for those having roles in embryonic development (Table S1), consistent with the concept that NuRD-mediated control of transcriptional heterogeneity is important for ESC lineage commitment and differentiation. Of these genes, approximately half (90 using ChIP-seq data from Reynolds et al., 2012, or 114 using ChIP-seq data from Whyte et al., 2012) have been shown to be direct Mi2β targets in wild-type ESCs, providing further evidence of NuRD-mediated transcriptional regulation.

This method of identifying candidate genes for NuRD-dependent transcriptional heterogeneity independently identified both *Zfp42* and *Klf4*. Notably, the set of genes also includes *Tbx3*, indicating that *Tbx3* does show heterogeneity in ESC populations (Figure 5A) at the transcript level, although we could not detect protein heterogeneity by antibody staining in wild-type cells (Figure 4A). Although correlation between protein and transcript abundance is highly dependent on the half-life of individual proteins, we were able to verify both NuRD-dependent protein heterogeneity for two additional genes from this list, *Zfp57* and *Esrrb* (Figure 5C), and direct binding to the respective promoters by Mbd3 and Mi2β by ChIP (Figure 5D). Both *Zfp57* and *Esrrb* are implicated in cell fate decisions during development (Chen et al., 2008; Li et al., 2008).

This analysis shows that a large number of genes that exhibit transcriptional heterogeneity are also regulated by NuRD. Because this approach will only identify those genes that are coregulated with *Zfp42* and will miss genes regulated by other mechanisms (such as *Klf5*; Hall et al., 2009), it will underestimate the total number of NuRD-regulated genes showing transcriptional heterogeneity in ESCs. Based on the extent of this effect, we propose that NuRD is generally important for the control of transcriptional heterogeneity in ESCs.

### A Balance between Activating and Silencing Activities Underlies Transcriptional Heterogeneity in ESC Populations

**Figure 6:**
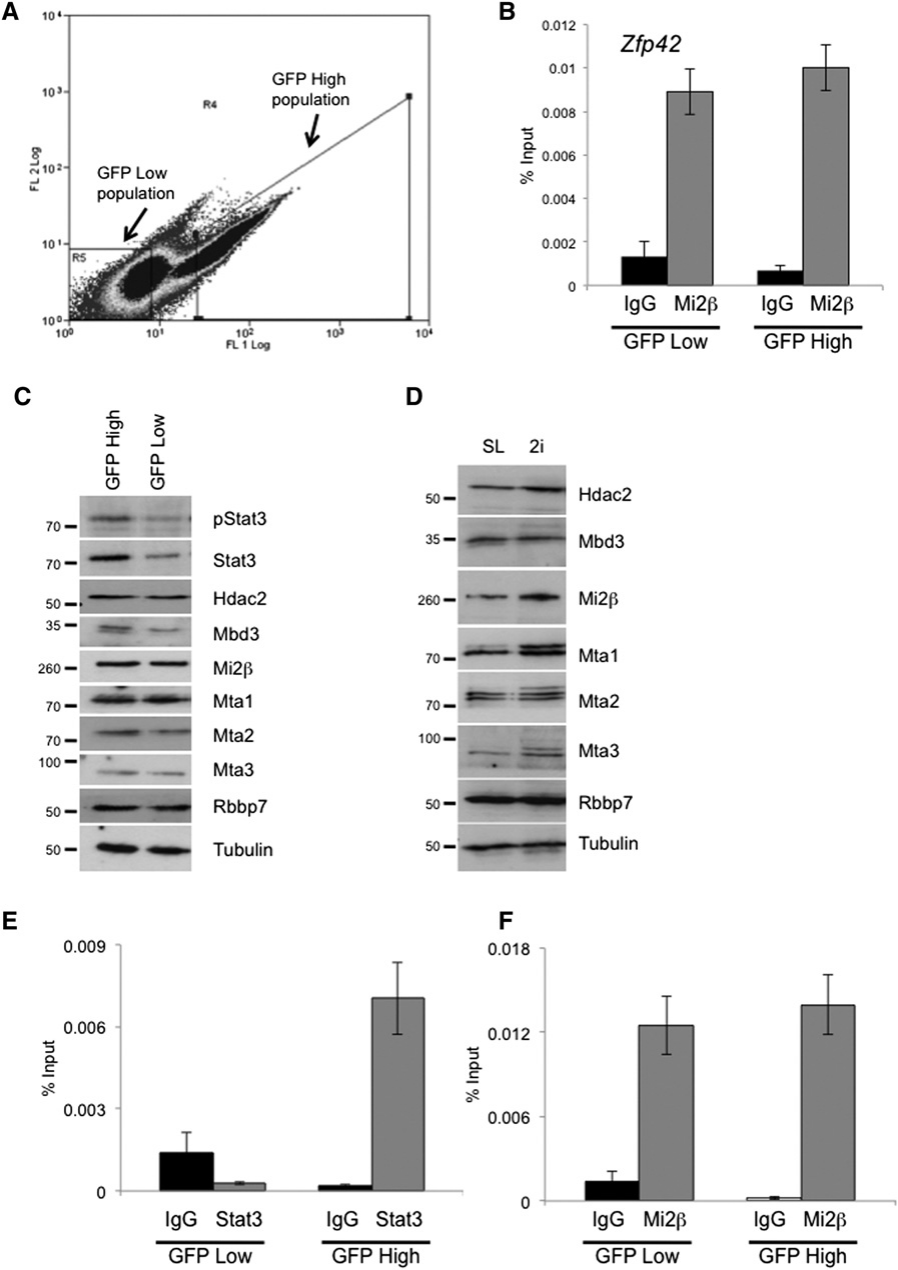
Variable Gene Expression Correlates with Variable Activator Activity (A) ESCs expressing GFPd2 from the *Zfp42* locus (Zfp42-GFPd2) were separated according to GFP intensity (x axis) and side scatter (y axis). Gate R5 contains the GFP-low sorted fraction and gate R4 contains the GFP-high fraction used for ChIP. (B) ChIP was performed using anti-Mi2β or a mouse IgG control antibody in Zfp42-GFPd2-low and Zfp42-GFPd2-high cells as shown in (A). (C) Western blots showing relative levels of Stat3 and phospho-Stat3 (pStat3) or indicated NuRD components in sorted Zfp42-GFPd2-high (GFP High) and Zfp42-GFPd2-low (GFP Low) cells. Protein sizes are shown at left in kDa. α-Tubulin is shown as a loading control. (D) Western blots for indicated NuRD components in ESCs maintained in serum and LIF (SL) or 2i/LIF (2i) conditions. Protein sizes are shown at left in kDa. α-Tubulin is shown as a loading control. (E and F) ChIP for Stat3 (E) or Mi2β (F) at the *Socs3* promoter as measured in Zfp42-GFPd2-low and -high populations.

For those genes exhibiting transcriptional heterogeneity in ESCs, NuRD is required both to generate the transcription-low cell populations and to limit the upper range of active transcription. However, this variability is unlikely to arise solely due to changes in the repressive activity of NuRD. Indeed, we found no evidence for variations in the level of Mi2β binding to the *Zfp42* promoter by ChIP in Zfp42-GFPd2-high and Zfp42-GFPd2-low ESC populations separated using flow cytometry (Figures 6A and 6B). Neither could we detect any evidence for variability in the abundance of NuRD component proteins between these two cell populations or between serum/LIF conditions and 2i/LIF conditions (Figures 6C and 6D). While neither of these are a direct measure of NuRD activity, transcriptional regulation is likely to involve a balance between both positive and negative influences. In fact, gene expression analysis (Table S1) and western blotting (Figure 6C) indicated a general reduction of Stat3 activity in the Zfp42-GFPd2-low ESCs. Consistent with these measurements, Stat3 could only be detected in association with the promoter of its target gene, *Socs3*, in the Zfp42-GFPd2-high ESCs (Figure 6E), while Mi2β was found to interact with the *Socs3* promoter equivalently in both populations (Figure 6F). Together these data indicate that the LIF/Stat3-mediated transcriptional activation of pluripotency genes varies from cell to cell within self-renewing ESC cultures, and that the interplay between this variable activation signal and the NuRD-mediated repressive effect produces transcriptional heterogeneity observed in ESCs cultured in serum and LIF conditions.

## Discussion

An emerging theme in stem cell biology is that seemingly homogeneous stem cell populations can be heterogeneous with respect to the abundance of certain transcripts and/or proteins (Huang, 2009; Enver et al. 2009; Raj and van Oudenaarden, 2008). While it is becoming increasingly clear that cellular heterogeneity may play an important role in stem cell differentiation, the mechanisms controlling transcriptional heterogeneity in mammalian ESCs have not yet been identified. Here we show that a chromatin-modifying corepressor controls both the transcriptional heterogeneity and the dynamic range of expression from a set of developmentally important genes in ESCs, and that these activities correlate with the ability of ESCs to exit self-renewal.

**Figure 7:**
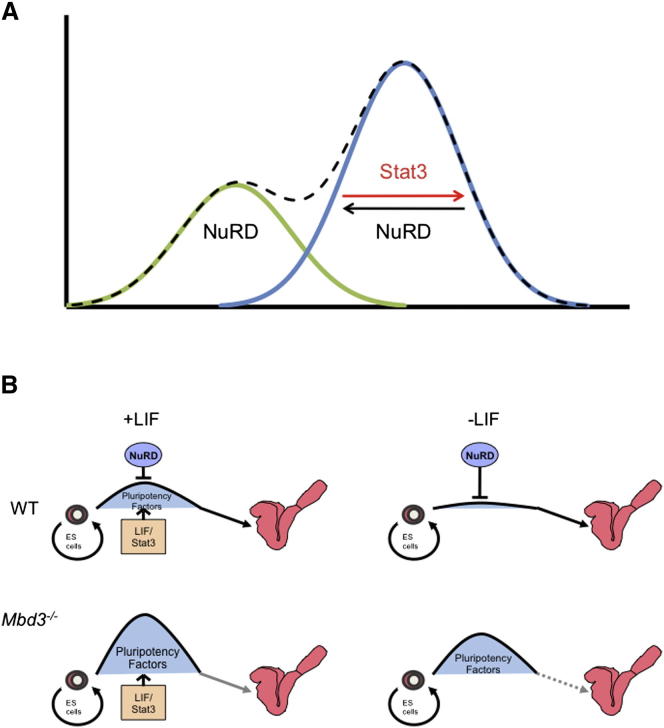
Models of NuRD Function in ESCs (A) NuRD function at the cell population level. Schematized graph of the protein distribution for pluripotency proteins in a population of self-renewing ESCs is shown as a dashed black line. This distribution is likely to be made up of two distinct subpopulations, represented by the green and blue curves. Within the protein-high (blue) population, Stat3 stimulates transcription while NuRD restricts expression levels. Self-renewing cultures also contain cells (green) in which Stat3 signaling is not active and NuRD-mediated repression is unopposed, resulting in the formation of a distinct subpopulation of cells in which expression of the gene is low or off. (B) NuRD function at the level of individual ESCs. ESCs are maintained in a self-renewing state (left-hand side), which is energetically favorable in cells grown in serum and LIF (or 2i) conditions. To exit self-renewal and contribute toward the somatic lineages (right-hand side) involves overcoming a differentiation barrier, the height of which is maintained by LIF/Stat3, which in turn promotes the expression of pluripotency factors. In contrast NuRD acts to restrict the height of this barrier by delimiting the expression levels of pluripotency factors. In *Mbd3^−/−^* ESCs the limiting effect of NuRD is gone, so the pluripotency factors are overexpressed, resulting in an increased height of the differentiation barrier, and ESCs cannot differentiate even upon LIF withdrawal.

NuRD is a well-characterized corepressor that has been shown to repress transcription in a variety of developmental contexts (McDonel et al., 2009). Here we show that NuRD associates with the promoters of several actively transcribed genes in ESCs, consistent with global profiles of NuRD complex component protein binding in mammalian cells (Reynolds et al., 2012; Wang et al., 2009; Whyte et al., 2012; Zhang et al., 2012). We further show that NuRD complex occupancy at some pluripotency-associated genes serves to both silence gene expression in a subpopulation of cells and limit transcription levels in the remaining cell population to within a range that can be responsive to the presence or absence of differentiation signals (Figure 7A).

We propose that these two different consequences, i.e., gene silencing and transcriptional modulation, both result from transcriptional damping activity of NuRD combined with the presence or absence, respectively, of transcriptional activation inputs provided by the self-renewal signaling cascade. In self-renewing cells, the silencing activity of NuRD at pluripotency-associated genes is counteracted by the activating downstream effects of LIF signaling and/or Erk and Gsk3 inhibition, resulting in moderate levels of transcription (Figure 7A). Upon removal or inactivation of self-renewal signals, the positive transcriptional effect is lost, and NuRD activity silences gene expression. In the absence of Mbd3, the NuRD complex fails to assemble, allowing increased gene expression in unstimulated cells and leaving the activating effect of self-renewal factors unopposed; both effects result in generally higher, more homogeneous transcript (and protein) levels. Upon LIF and/or 2i withdrawal, this stimulatory effect is removed, but in the absence of NuRD the gene cannot be silenced, resulting in failure of lineage commitment.

We propose a model in which ESCs maintained in self-renewing conditions face a barrier prohibiting differentiation (Figure 7B). The extent of this influence is defined by transcription of pluripotency genes and maintained by LIF and Stat3 signaling, but is limited by the activity of the NuRD complex. Withdrawal of LIF from ESC cultures under standard conditions results in an abrupt decrease in expression of pluripotency genes, lowering the barrier to differentiation and allowing cells to exit self-renewal. In the absence of Mbd3, NuRD is not able to limit expression of pluripotency genes, effectively raising the barrier to differentiation. In this scenario loss of LIF signaling (or of Erk and Gsk3 inhibition) results in a decrease in the transcript and protein levels of pluripotency factors, but this is not sufficient to allow cells to exit the self-renewal program. We propose that the effect of NuRD's modulatory activity in wild-type, self-renewing ESCs is to constrain the barrier to differentiation within a range that can be overcome when self-renewal signals are withdrawn (Figure 7B).

ESCs maintained in standard serum and LIF conditions exhibit expression level heterogeneity for a number of different transcription factors, and the status of a number of these genes has been functionally linked to the differentiation state of individual ESCs (Chambers, 2004; Hayashi et al., 2008; Toyooka et al., 2008). Cells grown in serum and LIF can differentiate: they spontaneously generate differentiated cells in culture because they are heterogeneous with respect to the expression of a variety of pluripotency-associated genes, and hence are heterogeneous in their immediate differentiation potential. In 2i/LIF conditions this transcriptional heterogeneity is largely suppressed, as is the ability of ESCs to spontaneously differentiate (Guo et al., 2010; Leitch et al., 2010).

Differentiation of ESCs in serum and LIF is believed to occur first in those cells expressing low levels of pluripotency genes, e.g., those falling within the smaller peak in Figure 7A (Chambers et al., 2007; Hayashi et al., 2008; Kalmar et al., 2009; Toyooka et al., 2008). Prior to lineage commitment, cells in the larger, protein-high peak must first reduce expression of pluripotency factors, resulting in a shift of the population toward low protein expression (Figures 4A and 4B). In contrast, cells maintained in 2i/LIF conditions all exist in the protein high peak, and more uniformly transit to a protein-low position as they exit self-renewal (Figures 4C and 4D). Here we show that the NuRD complex directly controls this transition out of the self-renewal state by enabling cells to extinguish expression of a number of pluripotency-associated genes. Additionally, NuRD functions to restrict the upper limits of gene expression (Figure 7A). Notably, we describe a model of transcriptional regulation in which a corepressor complex regulates gene expression, not only by straightforward silencing but also by restricting the dynamic range of transcription. By artificially reducing the expression levels of NuRD targets in *Mbd3^−/−^* ESCs, we were able to restore, to a moderate degree, their ability to engage in a developmental program. We conclude that the ability of ESCs to exhibit NuRD-dependent transcriptional heterogeneity for key proteins correlates with their ability to commit to differentiate.

## Experimental Procedures

A detailed description of the experimental procedures is provided in the Supplemental Information.

### ESCs

ESCs were grown in standard serum and LIF or 2i and LIF (Nichols et al., 2009) conditions. *Mbd3^−/−^* ESC lines have been described (Kaji et al., 2006). The MER-Mbd3b-MER-expressing ESC line has been described (Reynolds et al., 2012). To produce the Zfp42-GFPd2 allele (Wray et al., 2011) in *Mbd3^−/−^* and control cells, the coding region of *Zfp42* was replaced by a destabilized GFPd2 (Clontech) by homologous recombination in *Mbd3^Flox/−^* ESCs and in *Mbd3^−/−^* ESCs.

### ChIP

ChIP for endogenous proteins was carried out according to standard methods. Cells were fixed either with 1% formaldehyde for 10 min at room temperature or with disuccinimidyl glutarate (DSG) (Sigma) for 45 min prior to formaldehyde for Mi2β ChIP as described (Reynolds et al., 2012). ChIPs were performed a minimum of three times and qPCR was carried out in triplicate. ChIP using biotin-tagged Mbd3 or Mi2β was carried out as described (Kolodziej et al., 2009).

### Gene Expression Analyses

To visualize protein levels in cell populations, cells were grown, fixed, stained, and visualized in 96-well dishes. Staining intensity values were measured for Oct4-positive nuclei using Volocity software (Perkin Elmer) and were used to create frequency distribution plots. Data from at least three images taken from at least two different wells of cells were collated and processed together to generate each distribution. Graphs shown were made from one experiment but are representative of multiple independent experiments.

### mRNA Sequencing

Total polyA+ RNA was processed for library construction and sequencing according to standard methods. Sequencing was performed on the Illumina GAIIx yielding 38–41M single-end 105 bp reads per library. Sequences were aligned to the July 2007 assembly of the mouse genome (NCBI37/mm9).

mRNA sequence data obtained from Zfp42-GFPd2-high and Zfp42-GFPd2-low populations (Marks et al., 2012) were compared for expression level changes. Genes showing a 3-fold or greater difference in expression levels (and for which ≥10 unique reads could be mapped to the gene in the Zfp42-GFPd2-high population) were considered to show transcriptional heterogeneity.

## Accession Numbers

Sequencing data are available in the ArrayExpress repository under accession E-MTAB-997.

## Supplemental Information

Document S1. Figures S1–S3 and Supplemental Experimental Procedures

Table S1. Genes Showing Transcriptional Heterogeneity and NuRD-Dependent Transcription Levels, and Associated GO Terms (Related to Figure 5) The Excel document contains three tabs as follows: (1) A list of genes showing ≥3× expression differences between Zfp42-GFPd2-high and -low populations, and showing significant misexpression in *Mbd3^−/−^* ESCs; (2) a subset of genes listed in Tab 1 also associated with direct Mi2β binding by ChIP-seq as reported by Reynolds et al. (2012) or by Whyte et al. (2012); and (3) GO categories significantly overrepresented with the list of genes in Tab 1. Only those GO categories containing four or more genes in the list are shown.

## References

[bib1] Ahringer J. (2000). NuRD and SIN3 histone deacetylase complexes in development. Trends Genet..

[bib2] Chambers I. (2004). The molecular basis of pluripotency in mouse embryonic stem cells. Cloning Stem Cells.

[bib3] Chambers I., Colby D., Robertson M., Nichols J., Lee S., Tweedie S., Smith A. (2003). Functional expression cloning of Nanog, a pluripotency sustaining factor in embryonic stem cells. Cell.

[bib4] Chambers I., Silva J., Colby D., Nichols J., Nijmeijer B., Robertson M., Vrana J., Jones K., Grotewold L., Smith A. (2007). Nanog safeguards pluripotency and mediates germline development. Nature.

[bib48] Chen C., Sun X., Ran Q., Wilkinson K.D., Murphy T.J., Simons J.W., Dong J.-T. (2005). Ubiquitin-proteasome degradation of KLF5 transcription factor in cancer and untransformed epithelial cells. Oncogene.

[bib49] Chen Z.Y., Wang X., Zhou Y., Offner G., Tseng C.C. (2005). Destabilization of Kruppel-like factor 4 protein in response to serum stimulation involves the ubiquitin-proteasome pathway. Cancer Res..

[bib5] Chen X., Xu H., Yuan P., Fang F., Huss M., Vega V.B., Wong E., Orlov Y.L., Zhang W., Jiang J. (2008). Integration of external signaling pathways with the core transcriptional network in embryonic stem cells. Cell.

[bib6] Ema M., Mori D., Niwa H., Hasegawa Y., Yamanaka Y., Hitoshi S., Mimura J., Kawabe Y., Hosoya T., Morita M. (2008). Krüppel-like factor 5 is essential for blastocyst development and the normal self-renewal of mouse ESCs. Cell Stem Cell.

[bib50] Enver T., Pera M., Peterson C., Andrews P.W. (2009). Stem cell states, fates, and the rules of attraction. Cell Stem Cell.

[bib7] Guo G., Huss M., Tong G.Q., Wang C., Li Sun L., Clarke N.D., Robson P. (2010). Resolution of cell fate decisions revealed by single-cell gene expression analysis from zygote to blastocyst. Dev. Cell.

[bib8] Hall J., Guo G., Wray J., Eyres I., Nichols J., Grotewold L., Morfopoulou S., Humphreys P., Mansfield W., Walker R. (2009). Oct4 and LIF/Stat3 additively induce Krüppel factors to sustain embryonic stem cell self-renewal. Cell Stem Cell.

[bib9] Hayashi K., Lopes S.M., Tang F., Surani M.A. (2008). Dynamic equilibrium and heterogeneity of mouse pluripotent stem cells with distinct functional and epigenetic states. Cell Stem Cell.

[bib10] Huang S. (2009). Non-genetic heterogeneity of cells in development: more than just noise. Development.

[bib11] Kaji K., Caballero I.M., MacLeod R., Nichols J., Wilson V.A., Hendrich B. (2006). The NuRD component Mbd3 is required for pluripotency of embryonic stem cells. Nat. Cell Biol..

[bib12] Kaji K., Nichols J., Hendrich B. (2007). Mbd3, a component of the NuRD co-repressor complex, is required for development of pluripotent cells. Development.

[bib13] Kalmar T., Lim C., Hayward P., Muñoz-Descalzo S., Nichols J., Garcia-Ojalvo J., Martinez Arias A. (2009). Regulated fluctuations in nanog expression mediate cell fate decisions in embryonic stem cells. PLoS Biol..

[bib14] Kashiwagi M., Morgan B.A., Georgopoulos K. (2007). The chromatin remodeler Mi-2beta is required for establishment of the basal epidermis and normal differentiation of its progeny. Development.

[bib16] Kolodziej K.E., Pourfarzad F., de Boer E., Krpic S., Grosveld F., Strouboulis J. (2009). Optimal use of tandem biotin and V5 tags in ChIP assays. BMC Mol. Biol..

[bib17] Latos P.A., Helliwell C., Mosaku O., Dudzinska D.A., Stubbs B., Berdasco M., Esteller M., Hendrich B. (2012). NuRD-dependent DNA methylation prevents ES cells from accessing a trophectoderm fate. Biology Open.

[bib18] Leitch H.G., Blair K., Mansfield W., Ayetey H., Humphreys P., Nichols J., Surani M.A., Smith A. (2010). Embryonic germ cells from mice and rats exhibit properties consistent with a generic pluripotent ground state. Development.

[bib19] Li Y., McClintick J., Zhong L., Edenberg H.J., Yoder M.C., Chan R.J. (2005). Murine embryonic stem cell differentiation is promoted by SOCS-3 and inhibited by the zinc finger transcription factor Klf4. Blood.

[bib20] Li X., Ito M., Zhou F., Youngson N., Zuo X., Leder P., Ferguson-Smith A.C. (2008). A maternal-zygotic effect gene, Zfp57, maintains both maternal and paternal imprints. Dev. Cell.

[bib21] Marks H., Kalkan T., Menafra R., Denissov S., Jones K., Hofemeister H., Nichols J., Kranz A., Stewart A.F., Smith A., Stunnenberg H.G. (2012). The Transcriptional and Epigenomic Foundations of Ground State Pluripotency. Cell.

[bib22] Matsui Y., Zsebo K., Hogan B.L. (1992). Derivation of pluripotential embryonic stem cells from murine primordial germ cells in culture. Cell.

[bib23] McDonel P., Costello I., Hendrich B. (2009). Keeping things quiet: roles of NuRD and Sin3 co-repressor complexes during mammalian development. Int. J. Biochem. Cell Biol..

[bib24] Miccio A., Wang Y., Hong W., Gregory G.D., Wang H., Yu X., Choi J.K., Shelat S., Tong W., Poncz M., Blobel G.A. (2010). NuRD mediates activating and repressive functions of GATA-1 and FOG-1 during blood development. EMBO J..

[bib25] Murawska M., Hassler M., Renkawitz-Pohl R., Ladurner A., Brehm A. (2011). Stress-induced PARP activation mediates recruitment of Drosophila Mi-2 to promote heat shock gene expression. PLoS Genet..

[bib26] Nichols J., Silva J., Roode M., Smith A. (2009). Suppression of Erk signalling promotes ground state pluripotency in the mouse embryo. Development.

[bib27] Niwa H. (2007). How is pluripotency determined and maintained?. Development.

[bib28] Niwa H., Burdon T., Chambers I., Smith A. (1998). Self-renewal of pluripotent embryonic stem cells is mediated via activation of STAT3. Genes Dev..

[bib29] Niwa H., Ogawa K., Shimosato D., Adachi K. (2009). A parallel circuit of LIF signalling pathways maintains pluripotency of mouse ES cells. Nature.

[bib30] Nóbrega M.A., Zhu Y., Plajzer-Frick I., Afzal V., Rubin E.M. (2004). Megabase deletions of gene deserts result in viable mice. Nature.

[bib31] Raj A., van Oudenaarden A. (2008). Nature, nurture, or chance: stochastic gene expression and its consequences. Cell.

[bib32] Resnick J.L., Bixler L.S., Cheng L., Donovan P.J. (1992). Long-term proliferation of mouse primordial germ cells in culture. Nature.

[bib33] Reynolds N., Salmon-Divon M., Dvinge H., Hynes-Allen A., Balasooriya G., Leaford D., Behrens A., Bertone P., Hendrich B. (2012). NuRD-mediated deacetylation of H3K27 facilitates recruitment of Polycomb Repressive Complex 2 to direct gene repression. EMBO J..

[bib34] Takahashi K., Yamanaka S. (2006). Induction of pluripotent stem cells from mouse embryonic and adult fibroblast cultures by defined factors. Cell.

[bib36] Toyooka Y., Shimosato D., Murakami K., Takahashi K., Niwa H. (2008). Identification and characterization of subpopulations in undifferentiated ES cell culture. Development.

[bib37] Wang Z., Zang C., Cui K., Schones D.E., Barski A., Peng W., Zhao K. (2009). Genome-wide mapping of HATs and HDACs reveals distinct functions in active and inactive genes. Cell.

[bib38] Whyte W.A., Bilodeau S., Orlando D.A., Hoke H.A., Frampton G.M., Foster C.T., Cowley S.M., Young R.A. (2012). Enhancer decommissioning by LSD1 during embryonic stem cell differentiation. Nature.

[bib39] Wray J., Kalkan T., Smith A.G. (2010). The ground state of pluripotency. Biochem. Soc. Trans..

[bib40] Wray J., Kalkan T., Gomez-Lopez S., Eckardt D., Cook A., Kemler R., Smith A. (2011). Inhibition of glycogen synthase kinase-3 alleviates Tcf3 repression of the pluripotency network and increases embryonic stem cell resistance to differentiation. Nat. Cell Biol..

[bib41] Yamanaka S. (2009). A fresh look at iPS cells. Cell.

[bib42] Ying Q.L., Wray J., Nichols J., Batlle-Morera L., Doble B., Woodgett J., Cohen P., Smith A. (2008). The ground state of embryonic stem cell self-renewal. Nature.

[bib43] Yoshida T., Hazan I., Zhang J., Ng S.Y., Naito T., Snippert H.J., Heller E.J., Qi X., Lawton L.N., Williams C.J., Georgopoulos K. (2008). The role of the chromatin remodeler Mi-2beta in hematopoietic stem cell self-renewal and multilineage differentiation. Genes Dev..

[bib44] Zhang Y., Ng H.H., Erdjument-Bromage H., Tempst P., Bird A., Reinberg D. (1999). Analysis of the NuRD subunits reveals a histone deacetylase core complex and a connection with DNA methylation. Genes Dev..

[bib45] Zhang X., Zhang J., Wang T., Esteban M.A., Pei D. (2008). Esrrb activates Oct4 transcription and sustains self-renewal and pluripotency in embryonic stem cells. J. Biol. Chem..

[bib46] Zhang J., Jackson A.F., Naito T., Dose M., Seavitt J., Liu F., Heller E.J., Kashiwagi M., Yoshida T., Gounari F. (2012). Harnessing of the nucleosome-remodeling-deacetylase complex controls lymphocyte development and prevents leukemogenesis. Nat. Immunol..

[bib47] Zhu D., Fang J., Li Y., Zhang J. (2009). Mbd3, a component of NuRD/Mi-2 complex, helps maintain pluripotency of mouse embryonic stem cells by repressing trophectoderm differentiation. PLoS ONE.

