# Supplementary material for: NuRD Suppresses Pluripotency Gene Expression to Promote Transcriptional Heterogeneity and Lineage Commitment
Source: Cell Stem Cell. 2012 May 4;10(5):583–94. doi: 10.1016/j.stem.2012.02.020 (PMC3402183; doi:10.1016/j.stem.2012.02.020)
Supplement: Document S1. Figures S1–S3 and Supplemental Experimental Procedures [file mmc1.pdf]

Cell Stem Cell, volume *10*

Supplemental Information

NuRD Suppresses Pluripotency Gene Expression to Promote Transcriptional Heterogeneity and Lineage Commitment

Nicola Reynolds, Paulina Latos, Antony Hynes-Allen, Remco Loos, Donna Leaford, Aoife O'Shaughnessy, Olukunbi Mosaku, Jason Signolet, Philip Brennecke, Tüzer Kalkan, Ita Costello, Peter Humphreys, William Mansfield, Kentaro Nakagawa, John Strouboulis, Axel Behrens, Paul Bertone, and Brian Hendrich

Supplemental Experimental Procedures

ES Cell Culture

ES cells were grown in standard serum and LIF or 2i and LIF (2i = N2B27 medium + 1µM PD0325901 + 3µM CHIRON99021 (Nichols et al., 2009); inhibitors synthesised in the Division of Signal Transduction Therapy, University of Dundee, Dundee, UK) conditions, or grown in complete medium lacking LIF, or in N2B27 media lacking inhibitors and LIF to induce exit from self-renewal. Mbd3-null ES cell lines have been described (Kaji et al., 2006). The MER-Mbd3b-MER-expressing ES cell line has been described (Reynolds et al., 2012). To produce the Zfp42-GFPd2 allele (Wray et al., 2011) in Mbd3-null and control cells, the coding region of *Zfp42* was replaced by a destabilised GFPd2 (Clontech) by homologous recombination in *Mbd3*$^{Flox/-}$ ES cells and in *Mbd3*$^{-/-}$ ES cells.

An ES cell line that constitutively expressed the bacterial BirA ligase (Driegen et al., 2005) was obtained from Dies Meijer (ErasmusMC, Rotterdam). Expression constructs in which either *Mbd3b* or *Chd4* cDNAs were fused with a biotinylation signal (Avi-tag) (de Boer et al., 2003) were then stably integrated into the BirAE5 ES cell line under the control of a constitutive promoter. Resulting cell lines were analysed for expression and biotinylation of the Avi-tagged proteins using the following antibodies: anti-Mbd3 (sc-9402, Santa Cruz Biotechnology), anti-Mi2β (a

gift from Katia Georgopoulos, Boston), anti-alpha tubulin (sc-5286, Santa Cruz Biotechnology) (Supplemental Fig. 1 and data not shown).

For clonal differentiation assays ES cells were plated on a 6-well gelatinized dish at the density of 600 cells per well in ES+LIF media and left in culture overnight. The cells were washed in PBS and culture continued for five days either in the presence or absence of LIF. The cells were stained using the Leukocyte Alkaline Phosphatase Kit (Sigma) and counted blind to the ES cell genotypes.

ES cell chimaeras were made by morula aggregation using standard procedures. ES cells were derived from the M3ß6C ES cell line (Kaji et al., 2006) which expresses LacZ from the *Mbd3* locus in all embryonic tissues. LacZ staining was used to monitor ES cell contribution to chimaeras. Embryos were dissected in PBS and fixed in PBS containing 1% paraformaldehyde and 0.5% glutaraldehyde for 0.5 to 2 hours at 0°C. Embryos were subsequently rinsed 3 times in 0.1 M NaPi pH 7.0, 2 mM $MgCl_2$, 0.02% NP40, 0.01% deoxycholate, and then stained overnight at 37°C in rinsing buffer plus 1 mg/ml Xgal, 5 mM $K_3Fe(CN)_6$, 5 mM $K_4Fe(CN)_6$, 1 mM EGTA. Scoring of embryos for ES cell integration was performed blind to the ES cell genotypes.

All mice were housed under standard conditions. All procedures were covered by a license granted by the UK Home Office and were approved by institutional ethics committees.

Chromatin Immunoprecipitation

Chromatin immunoprecipitations for endogenous proteins were carried out according to standard methods. In brief, cells were grown in normal ES cell media to approximately 50% confluency, after which incubation was continued for a further 24 hours either in the continued presence or absence of LIF. A sample of the culture was retained for RNA extraction and analysis of gene expression by quantitative RT-PCR. Cells were fixed either in 1% formaldehyde for 10 minutes at room temperature, or with DSG (disuccinimidyl glutarate (Sigma)) for 45 minutes prior to

formaldehyde for Mi2β ChIP as described (Reynolds et al., 2012). Anti-Mbd3 ChIP was performed according to the Myers Lab Protocol (http://myers.hudsonalpha.org/documents/Myers%20Lab%20ChIP-seq%20Protocol%20v041610.pdf). Chromatin was sheared by sonication (Bioruptor (Diagenode)) resulting in an average fragment size of approximately 300bp. Immunoprecipitations were carried out using an IgG control, anti-Mi2β (Abcam ab70469) (Reynolds et al., 2012), anti-Mbd3 (A302-528A, Bethyl Laboratories; Figure S1D), anti-Stat3 (sc-482, Santa Cruz Biotechnology) or anti-ERα (sc-543, Santa Cruz Biotechnology) antibody. For ChIP from cells grown in 2i/LIF conditions, cells were grown in 2i/LIF media for two passages and then processed as above. Quantitative PCR was carried out with gene specific primers listed below, or with TaqMan probes (Life Technologies). Chromatin IPs were performed a minimum of three times and qPCR carried out in triplicate. ChIP using biotin-tagged Mbd3 or Mi2β was carried out as described (Kolodziej et al., 2009) and analysed with gene specific primers listed below.

The percentage of total input DNA associated with immunoprecipitated proteins was calculated using standard procedures (see http://www.invitrogen.com/site/us/en/home/Products-and-Services/Applications/epigenetics-noncoding-rna-research/Chromatin-Remodeling/Chromatin-Immunoprecipitation-ChIP/chip-analysis.html)

microRNA constructs and knockdown ES cells

The microRNA sequences were designed using BLOCK-iT™ RNAi Designer (Life Technologies) and originally cloned into the pcDNA6.2™ 6.2-GW/EMGFP-miR vector (Life Technologies) and then re-cloned into a PiggyBac (PB) vector (Wang et al., 2008), using Gateway® cloning system (Life Technologies). The sequences targeted by the microRNAs are as follows:

Klf4-1: 5'- gatgggcaagtttgtgctgaa -3'

Klf4-2: 5'- ccaccttgccttacacatgaa -3'

Klf5-1: 5'- tgagaactggcctctacaaat -3'

Klf5-2: 5'- ccaaatttacctgccactctg -3'

LacZ: 5'-gactacacacaaatcagcgattt-3'

Scrambled: 5'-gtctccacgcgcagtacatttc-3'

To establish knock-down lines, 10^6 *Mbd3*$^{-/-}$ ES cells were co-transfected using Lipofectamine 2000 (Life Technologies) with 1 µg of pPB-Klf4 or 5-microRNA-ires-Hyg or control vector pPB-scrambled or LacZ-microRNA-ires-Hyg plus 2-3 µg of the PBase-expressing vector pCAGPBase (Wang et al., 2008). Stable transfectants were selected for hygromycin resistance. Rescue lines were made by expressing a cDNA in which conservative mutations were introduced into the microRNA target sequence. To produce such cDNAs a Phusion (New England Biolabs) site-directed mutagenesis protocol was used together with the following oligos:

Klf4-A: 5'-gggctgatgggcaagtttgtcttaaaggcgtctctgaccacccc-3'

Klf4-AR: 5'-gccacctggcggctgaggctgctgtggcgg-3'

Klf5-B: 5'-atcactcacctgagaactgggttgtacaaatcccagagaccatg-3'

Klf5-BR: 5'-gtcagggaggaagacgttcatgttgatgct-3'

Rescuing cDNAs were then cloned into the pCAGA3xFiP vector, transfected into knockdown cell lines, and stable transfectants selected for puromycin resistance.

Gene expression analyses

Total RNA was prepared using TRIzol reagent (Life Technologies) and treated with DNaseI (Promega). First-strand cDNA was synthesized using Superscript III reverse transcriptase (Life Technologies). Quantitative PCR was performed using Fast SYBR green Master Mix (Life Technologies) or TaqMan reagents (Life Technologies). Gene expression was determined relative to *Gapdh*, *ß-actin* or Ppia using the ΔCt method. All quantitative PCR (qPCR) reactions were performed in a 7900HT Fast Real-Time PCR System (Life Technologies) or a StepOne Real Time PCR System (Applied Biotechnologies). Sequences of the QPCR primers are listed

below. Expression of genes not listed below was tested using TaqMan assays (Applied Biosystems).

To visualise protein levels in cell populations, cells were grown on gelatin- or laminin-coated dishes and then fixed with 4% paraformaldehyde for 20 minutes, permeablised with 0.1% Triton X-100 in PBS for five minutes, and then blocked with 3% donkey serum in PBS. Antibody staining was performed in blocking solution using the following antibodies: anti-Oct4 (1/100, sc-5279 and sc-8628, Santa Cruz Biotechnology), anti-Nanog (1/250, ab21603, Abcam and RCAB0002P-F, Cosmo Bio Co), anti-Klf4 (1/250, AF3158, R&D Systems), anti-Klf5 (1/1000, J. Whitsett, Cincinatti, OH) (Wan et al., 2008), anti-Zfp57 (1/1000, sc-169866, Santa Cruz Biotechnology), anti-Esrrb (1/500, PP-H6705-00, R&D Systems) and anti-Tbx3 (1/250, sc-17871, Santa Cruz Biotechnology). Alexafluor-conjugated secondary antibodies (Life Technologies) were applied at 1/1000 in blocking solution. Cells were imaged using a Zeiss microscope and staining intensity values were measured for Oct4-positive nuclei using Volocity software (Perkin Elmer). Data from at least three images taken from at least two different wells of cells were collated and processed together to generate each distribution. Frequency distribution plots and were produced and statistics calculated using Microsoft Excel. Graphs shown were made from one experiment but are representative of multiple independent experiments.

RNA processing and library construction

Total RNA was extracted using the TRIzol method followed by treatment with TURBO DNase (Ambion). Polyadenylated transcripts were selected from 2 µg total RNA using Sera-Mag beads (Thermo Scientific). Between 60 and 100 ng mRNA was then sheared to approximately 200 nt fragments by focused ultrasound on the Covaris S2 using the following parameters: Duty Cycle = 10%, Intensity = 5, Cycles Per Burst = 200, for 75s with frequency sweeping enabled. First-strand cDNA synthesis was performed at 50°C for 2 hours using SuperScript III (Invitrogen) and random hexamer primers, followed by second-strand synthesis with DNA

Polymerase I at 16°C for 2 hours in the presence of RNaseH. End repair of double-stranded cDNA products was carried out with T4 DNA polymerase and T4 polynucleotide kinase (New England Biolabs). Blunted, phosphorylated cDNA fragments were then 3'-adenylated via Klenow fragment and ligated to sequencing adapters (Illumina) by T4 DNA ligase at 20°C for 30 minutes. PCR amplification of library constructs was carried out with Phusion DNA polymerase (Finnzymes) for 13 cycles. Purification of reaction products between each step was performed with Ampure XP paramagnetic beads (Beckman Coulter). Prior to sequencing, the molarity and size distribution of the libraries was assessed by DNA 1000 microfluidic chips on the Agilent 2100 Bioanalyzer.

Sequencing and data analysis

Sequencing was performed on the Illumina GAIIx yielding 38-41M single-end 105bp reads per library. Sequences were aligned to the July 2007 assembly of the mouse genome (NCBI37/mm9) using GSNAP (Wu and Nacu, 2010), where annotated splice junctions were provided from Ensembl Build 63 (Flicek et al., 2011) and up to five mismatches were allowed. Transcript quantification was performed using htseq-count, part of the HTSeq package for the R statistical computing platform (http://www-huber.embl.de/users/anders/HTSeq/doc/overview.html). Differentially expressed genes were identified with the Bioconductor package DESeq (Anders and Huber, 2010), and Gene Ontology (GO) enrichment analysis was performed using GOstats (Falcon and Gentleman, 2007). The reported *P*-values were corrected for multiple testing using the Benjamini & Hochberg method (Benjamini and Hochberg, 1995).

mRNA sequence data obtained from Zfp42-GFPd2-high and Zfp42-GFPd2-low populations (Marks et al. in revision) were compared for expression level changes. Genes showing a three-fold or greater difference in expression levels (and for which ≥10 unique reads could be mapped to the gene in the Zfp42-GFPd2-high population) were considered to show transcriptional heterogeneity.

Expression primers

Gene	Forward	Reverse
ß actin	GTGGGCCGCTCTAGACACCA	CGGTTGGCCTTAGGGTTCAGGGGG G
Klf2	CTAAAGGCGCATCTGCGTA	TAGTGGCGGGTAAGCTCGT
Klf4	GAGTTCCTCACGCCAACG	CGGGAAGGGAGAAGACACT
Klf5	CCGGAGACGATCTGAAACAC	CAGATACTTCTCCATTTCACATCTTG
Nanog	ATGCCTGCAGTTTTTCATCC	GAGGCAGGTCTTCAGAGGAA
Pou5f1	GGAAAGCAACTCAGAGGGAA	TTCTAGCTCCTTCTGCAGGG
Socs3	GCCTCGCCTCGGGGACCATA	CGGAGCCAGCGTGGATCTGC
Sox2	GCGGAGTGGAAACTTTTGTC	TATTTATAATCCGGGTGCTCCTT
Tbx3	GAACCTACCTGTTCCCGGAAA	CCATTGCCAGTGTCTCGAAAAC
Zfp42	AGTGTGCAGTGCAGCCAG	TGCTTTCTTCTGTGTGCAGG

Chromatin IP primers

Gene	Forward	Reverse	Distance from TSS/genomic position
Esrrb	ACCAGGTTCCCCTGAACGGT	GGGCCTCACCAATGAGGCAGA	-1.2kb
Esrrb	CTTTGGGACAGGCACACGGT	CGAGGGTGGCCAGGTCTGTAG	-0.19kb
Esrrb	CGTGCCGCAAGAGCTACGAG	CCCACCACGCACCTTGAATGG	0.35kb
Esrrb	TGGGCTAACCCACCAGGCTT	GAGGCAGCACACCCACGTTT	1.6kb
Esrrb	GGGCCTCTGAGATTGCCTAGTGT	CACCACGTGGGTCACTGGGA	2.2kb
Klf4	GGCCTGGCCTTGTAGTCCCCA	CCTCAGGAGGGCACTGTGGAA	-6kb
Klf4	GGCAGCAGATACCCAAAAGC	AAGCTAGGCAGAGGAAAGGG	-3.1kb
Klf4	CCTGTTGTCACACCCGGCAC	AGCAGGCAACACACGGGTTC	-2.3kb
Klf4	CTCACCCCCACCCTACG	ATTATCCGCGTGACTCATCC	-0.6kb
Klf4	ACACACACACACCCCCAAC	GGCCACGGTCGGTTC	-0.4kb
Klf4	GCCTGGCTGGCGTCACGG	CCACTGCGCGTTGCCATGGTA	-0.2kb
Klf4	TCGTTGACTTTGGGGCTCAGGTA	TCCGAAGCTTCCCTCCAATCCTC	0.58kb
Klf4	CTCTACAGCCTTCCGAGGTG	AAGACTAGTGGGGAACCGCT	1.0kb
Klf4	AGGAGGGCAGCAAACAGGCG	CAGGCTAGGAGGGGCAGGCA	2.9kb
Klf5	TGGGGGAGGGACAGAGACCCA	GGGAGCAGAGGCCCACAGGT	-6.4kb
Klf5	TTCCCTGACGGCAGCAGCAC	CCCCAGGCAGGTCCGCAATG	-4.1kb
Klf5	ACCAGGCCCAGGCAGGTTCA	ACCCCACCAAGCTGCGTGAC	-2.1kb
Klf5	AAACTCCATTCCCCGCCCCT	AACAGCCAGCTCAACCGGGTA	-0.87kb
Klf5	TACCCGGTTGAGCTGGCTGT	CTCCTTCGCCGAATGTGGGG	-0.78kb
Klf5	TTCCCCACATTCGGCGAAGGA	AAACAAGGGGCGGTGCGAAA	-0.62kb
Klf5	GTCGGAGGCGGGACCTCGTG	GCGTACCTGCCCCGCGACTA	-0.16kb
Klf5	GTCGGAGGCGGGACCTCGTG	GCGTACCTGCCCCGCGACTA	-0.06kb
Klf5	TTGCCATTGGCCACAACGCT	TACCTGCCCCGCGACTACTG	0.04kb
Klf5	CCGTGTCCCGCTCCCGTAATC	CCGCTCTGCTTACCTGGACCAGC	0.4kb
Klf5	GAGGAGCTGGTCCAGGTAAGCAGA	CAAGCAAAGAGAAGGTGCACGC	0.64kb
Klf5	TCCTCTCGGAGTCGCGTTGA	CGTGCGTGGGCGAAAGAAAC	1.1kb

Klf5	AGCATTGGCCCACGGAGAGGA	CTCCCGTGGGTGGGGAGAGG	1.9kb
Klf5	AGGGTGAAAGAGCTCGGCCA	GCCTCTCAGACAAGCCAAGGC	5.3kb
Socs3	CAGTGCAGAGTAGTGACTAAACATTAC	AAGGACCTGGGCGAGGGACC	0.8kb
Tbx3	GCCAGGACATCAGGCTCCCA	GTCGGGGGCCTGAGGATGAA	-3.3kb
Tbx3	GGCGCCTTCCCGTTTCTCTG	AAATGTGGGGGTGGGTGGGT	-2.8kb
Tbx3	AGGCACCCAGAGATAAGTGTGATG	GAGCTGCCGCTCTGGGCTTG	-0.7kb
Tbx3	GCCGCGGGCTTGAACTGTAG	ACGTCTGAAAGGCCTCAGCCA	-0.08kb
Tbx3	TGGATGGCTGAGGCCTTTCAGAC	AACTGGAACGTCGCTGCGC	0.08kb
Tbx3	GACGCCTCCTGCGAGAAAGC	TGTCCTGGTGCGTGGAGTGT	0.975
Tbx3	AGCTCTGGCCAAGCCCATCA	CAGATGTGCCTGGGGTCCCA	1.5kb
Tbx3	AGACCGCGCACTCGGCTTCT	TCCCGGGTCTCCCACTGCTG	2.1kb
Tbx3	GTGCCCTTGCCTGACCGAAAT	GGAAGGAGACCGAAAGTGGGGT	2.5kb
Tbx3	TCGCCAGCCTCAGTCCATAAGT	GGATTTTCCACCAGCTGGCTGA	2.9kb
Tbx3	GCCGGACCTGGAAGTGTGGA	GCCTCCTCGGAAACAACATGGT	4kb
Zfp42	GCAAATCACTGGCAGGGTATCCGA	CCTAGAGACTTCAGCCATGGCCT	-4.0kb
Zfp42	CGCCGGCTGCCAATGCATTTT	TCCCCGGAAGTGAGTTTGTGGCT	-1.7kb
Zfp42	TTACAGCCCAGGCTAAGGCTCTG	GTGAGGCCCTGTTTCAGTTTCCT	-1.4kb
Zfp42	CGCCGGCTGCCAATGCATTTT	TGTGGCTGAAGACAGAACAAGGAAC	-0.9kb
Zfp42	TTCCGGGGATGACAGGAGGT	ACGCCGGGCACCATTAAGAC	-0.75kb
Zfp42	GCTGCATGACCCACGCTCTCAAA	TCTCCCGGACCCCGCTACAAAG	-0.5kb
Zfp42	AGCTCTGGGTGGGTCACCTGAAG	TAGGCGGCTAGGAGTTCAGCTCC	-0.2kb
Zfp42	AGAGCGCATCGCATCGCTGT	TCAAACCCTTCCCTCCTCCCTTTC	TSS
Zfp42	GAGCGCATCGCATCGCTGTGG	CCCTTCCCTCCTCCCTTTCCTCATA	0.16kb
Zfp42	CCTTGGTCCATTGGCCATCACG	TAGGTGCCCTGTTACCTCGCT	0.365kb
Zfp42	GCAACGCAAATGGCTTTCCGT	TGGTCGGTAGTATGGCGGCC	1kb
Zfp42	GCGGGGAAAGATCGACTGCTTCT	CCGTCCCCGGAGAGGTGAGAT	1.4kb
Zfp42	GTCTTAATGGTGCCCGGCGT	AGCGTGGGTCATGCAGCGTT	3.0kb
Zfp57	ACTGTGTGCTGGGGCTACCT	TGGAGCCATGCTGACCTTGA	-1.3kb
Zfp57	GGCTGCCTGAGACCTGGGTT	GGTATCACATCACGGTCAAGTCGT	-0.68kb
Zfp57	AGCGCAGCTGAGACCAGTGA	GCTCCGTCCGCAGGTAACAC	-0.01kb
Zfp57	ACACCTGGGTGGGAGAAGCC	AGCCCCGGTCCCAGACTCTA	0.36kb
Zfp57	GGACCTGACCTCCGGCCAAT	TGGTGCAGGAGCCCTGAGAA	0.74kb
Zfp57	ACTGGACTCACGCTGCCAAGA	AGCCTGTGACTACTACCCAGCAT	1.5kb
Chr19 GFR	AGTCCCAATGGCAACTCCTCTACTT	GCAGTGCCAAAGCAGCAGTCT	chr19: 29002906
Chr19 GFR	CCCAGGAGGATCTGGAAGCCAAA	CTCCCAAGAAGAGCACACTTCCC	chr19: 29003306
Chr19 GFR	TTGCATATGGTTTCCAAGCCCCTTT	GGAGCATCCTTTGCTGAGACTGGA	chr19: 29003706
Chr19 GFR	GCTTCCCATGAGCCAGAGTGC	ACAGGTTCACCAATGGGGGACTAA	chr19: 29003906
Chr19 GFR	GGTTTGGCCTGTGGAACGGAT	GGACCACTTAGACCAGCGTGC	chr19: 29004606
Chr19 GFR	CTTGGGCATGTCCCTAGCCTTATCT	AGTGCCACGTCTTTTACCATGGAG	chr19: 29005106
Chr19 GFR	CCGAGTCGCAAGGCTGTCTTC	CTGACACGGGGCATGGACAC	chr19: 29005506
Chr19 GFR	ATTTGCTGGTGTCCATGCCCC	AGGAAGAGGCCTGGGGTTGAA	chr19: 29005606

Supplemental Figures

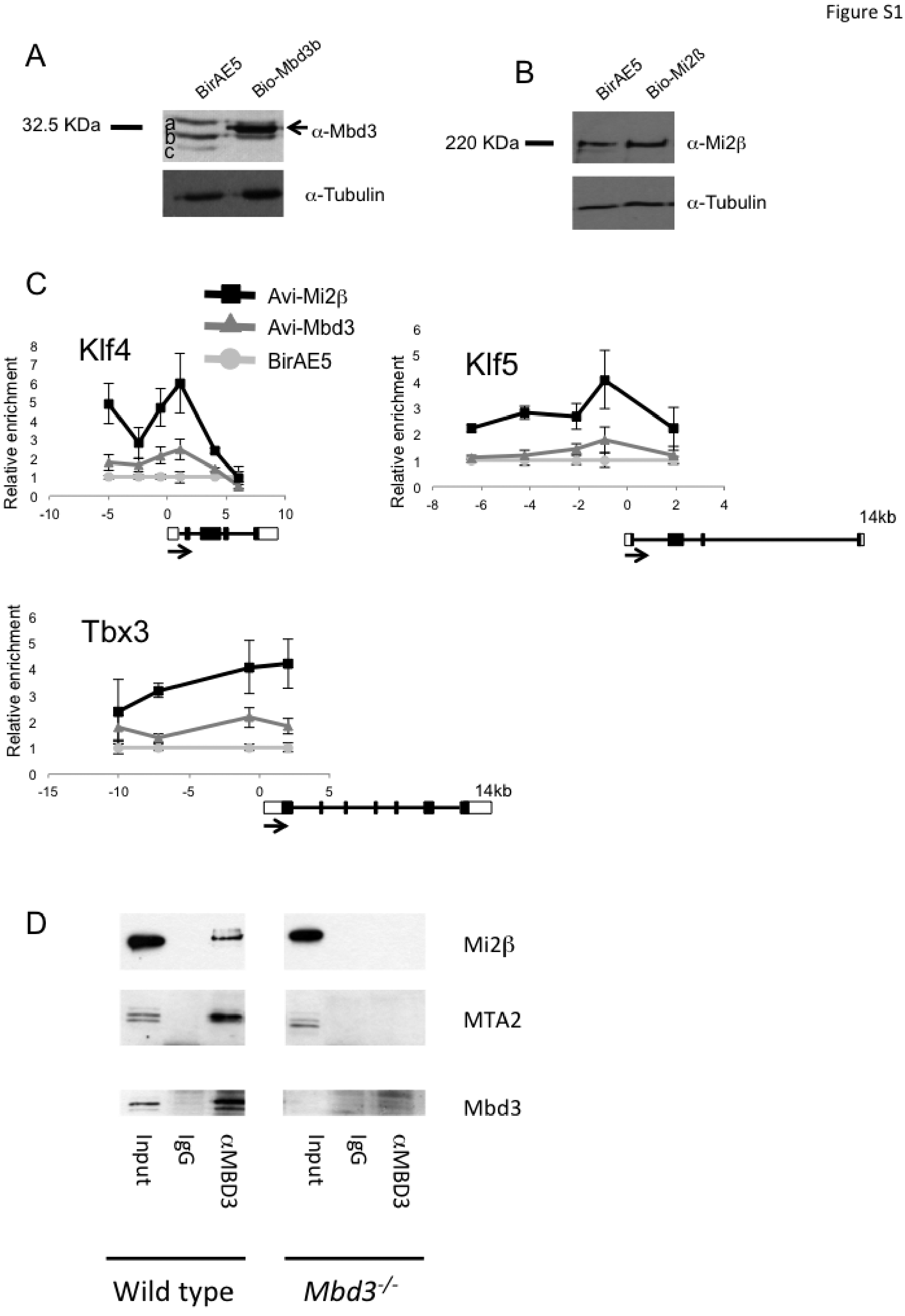

Supplemental Figure 1. Avi-ChIP of Mbd3b and Mi2β and validation of anti-Mbd3 antibody, related to Figure 1. Western blot analysis of BirAE5 ES cells stably transfected with Avi-tagged Mbd3b (A) or Avi-tagged Chd4 (B) using indicated

antibodies. Approximate sizes are indicated at left in kilodaltons. Mbd3 isoforms a, b, and c are indicated on the BirA western blot. Avi-Mbd3b is visible as a size-shifted protein relative to endogenous Mbd3b, while no size shift is visible for Avi-Mi2β. Endogenous Mbd3 proteins appear less abundant in the transfected cell lines. Blots were stripped and re-probed with an anti-alpha tubulin antibody as a loading control (lower panels). (C) Streptavidin-ChIP was performed in ES cells expressing BirA biotin ligase only (BirAE5, light grey lines), BirA and Avi-tagged Mbd3b (Avi-Mbd3b, dark grey lines), or BirA and Avi-tagged Chd4 (Avi-Mi2β, black lines) and plotted as enrichment relative to BirA (y-axis). Error bars represent s.e.m. Numbers along the x-axis indicate distance relative to transcription start site for indicated genes. D. Validation of the anti-Mbd3 antibody. Immunoprecipitates obtained using anti-Mbd3 or control IgG antibodies in wild type or $Mbd3^{-/-}$ ES cells was blotted and probed for proteins indicated at right.

Figure S2

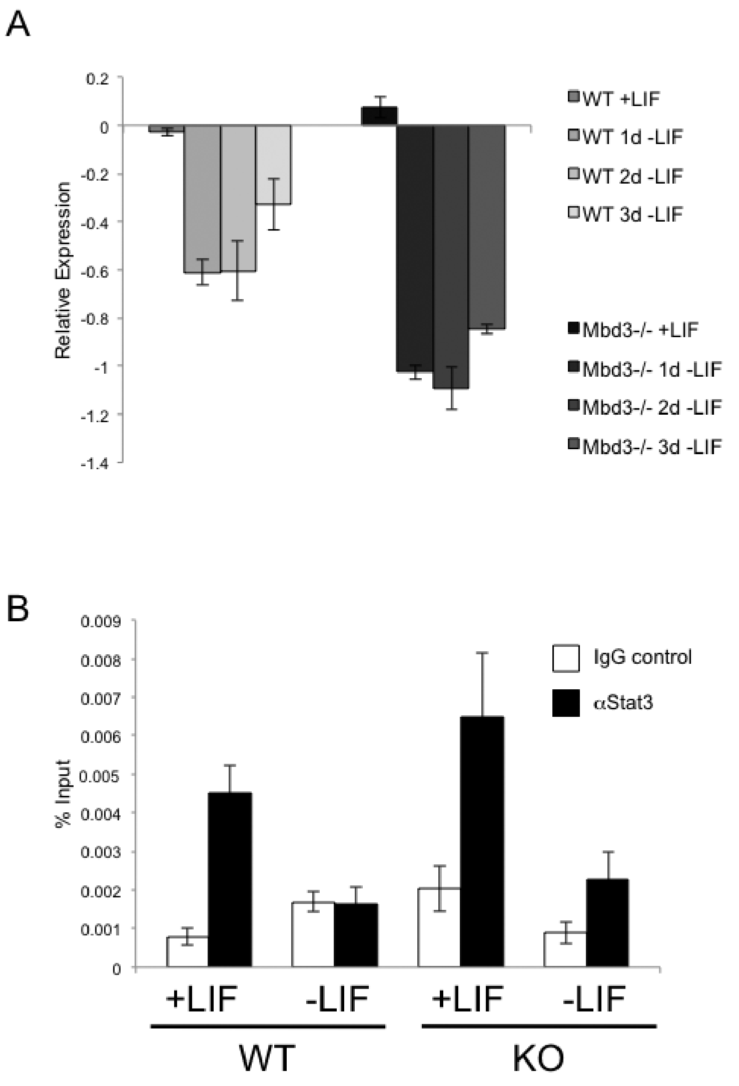

Supplemental Figure 2. Loss of Stat3 binding to *Socs3* upon LIF withdrawal, related to Figure 3. (A) Expression of *Socs3* in wild type and *Mbd3*$^{-/-}$ ES cells in LIF or in the absence of LIF for the indicated times is plotted relative to expression in wild type cells prior to LIF withdrawal. Error bars represent s.e.m. from multiple experiments performed on different wild type and mutant ES cell lines. (B) Chromatin immunoprecipitation for Stat3 (black bars) or control mouse IgG (white bars) at the *Socs3* promoter in wild type (WT) or *Mbd3*-null (KO) ES cells grown in self-renewing conditions (+LIF) or after 24 hours of LIF withdrawal (-LIF), and plotted as percentage of input (y-axis). Error bars represent s.e.m.

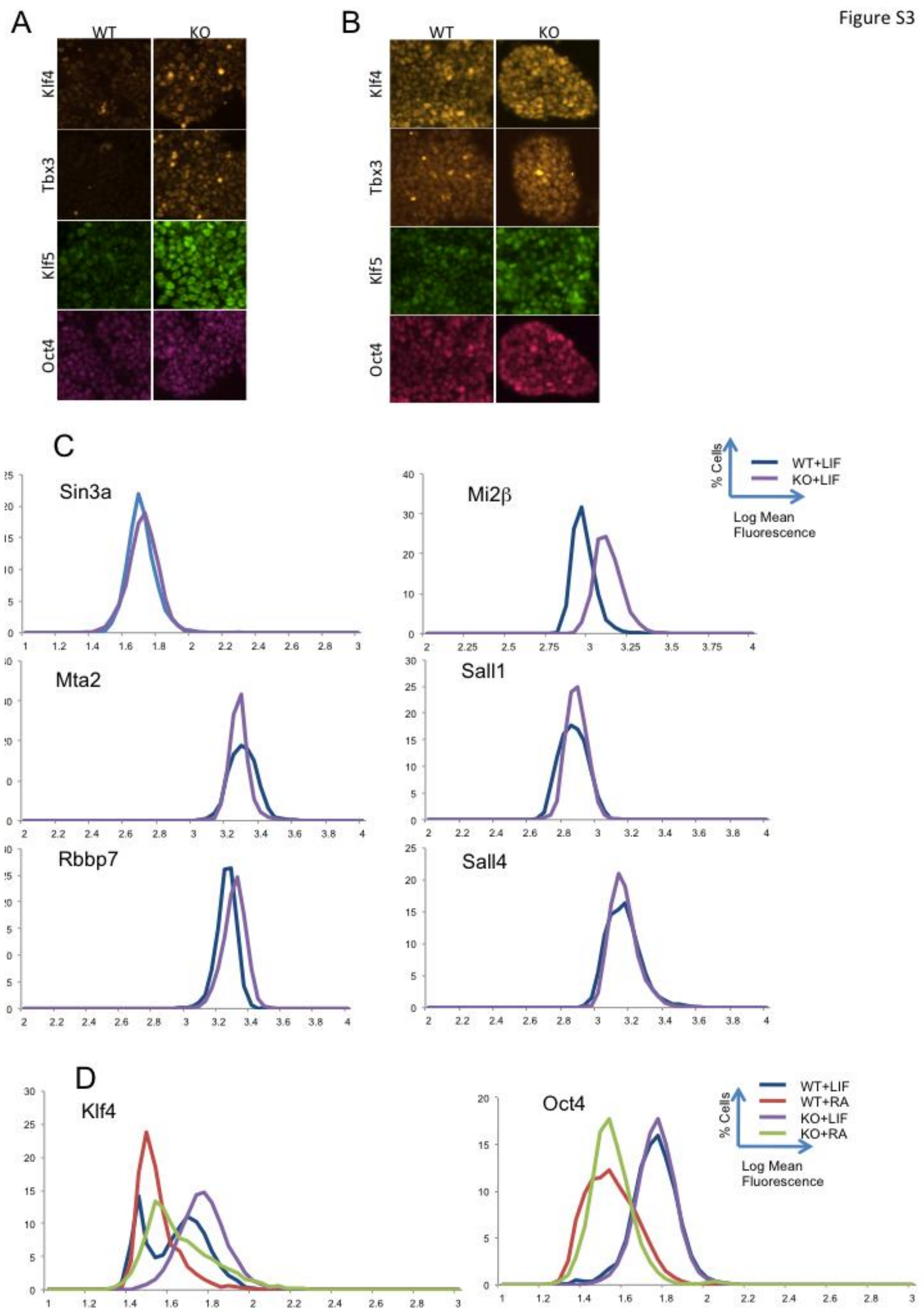

Supplemental Figure 3. Immunofluorescence analysis in *Mbd3*$^{-/-}$ ES cells, related to Figure 4. Immunofluorescence for indicated proteins in wild type (WT) or *Mbd3*$^{-/-}$ (KO) ES cell lines grown in standard serum and LIF conditions (A) or in 2i conditions (B). (C) Expression levels of indicated proteins were measured in wild

type (WT) and *Mbd3*$^{-/-}$ (KO) ES cell cultures by antibody staining and immunoflourescence microscopy. Relative fluorescence is plotted along the x-axis, with the proportion of cells indicated along the y-axis. N ≥ 5000 for all samples. (D) Expression levels for Klf4 and Oct4 were measured as in (C). Data is shown for wild type ES cells in self-renewing conditions (i.e. WT+LIF, blue line), wild type ES cells exposed to retinoic acid in the absence of LIF for 24 hours (WT+RA, red line), Mbd3-null ES cells in self-renewing conditions (KO+LIF, purple line) and Mbd3-null cells exposed to retinoic acid in the absence of LIF for 24 hours (KO+RA, green line); N > 4000 for all samples.

Supplementary References

Anders, S., and Huber, W. (2010). Differential expression analysis for sequence count data. Genome Biol *11*, R106.

Benjamini, Y., and Hochberg, Y. (1995). Controlling the False Discovery Rate: A Practical and Powerful Approach to Multiple Testing. Journal of the Royal Statistical Society Series B (Methodological) *57*, 289-300.

de Boer, E., Rodriguez, P., Bonte, E., Krijgsveld, J., Katsantoni, E., Heck, A., Grosveld, F., and Strouboulis, J. (2003). Efficient biotinylation and single-step purification of tagged transcription factors in mammalian cells and transgenic mice. Proceedings of the National Academy of Sciences of the United States of America *100*, 7480-7485.

Driegen, S., Ferreira, R., van Zon, A., Strouboulis, J., Jaegle, M., Grosveld, F., Philipsen, S., and Meijer, D. (2005). A generic tool for biotinylation of tagged proteins in transgenic mice. Transgenic Res *14*, 477-482.

Falcon, S., and Gentleman, R. (2007). Using GOstats to test gene lists for GO term association. Bioinformatics *23*, 257-258.

Flicek, P., Amode, M.R., Barrell, D., Beal, K., Brent, S., Chen, Y., Clapham, P., Coates, G., Fairley, S., Fitzgerald, S.*, et al.* (2011). Ensembl 2011. Nucleic acids research *39*, D800-806.

Kaji, K., Caballero, I.M., MacLeod, R., Nichols, J., Wilson, V.A., and Hendrich, B. (2006). The NuRD component Mbd3 is required for pluripotency of embryonic stem cells. Nat Cell Biol *8*, 285-292.

Kolodziej, K.E., Pourfarzad, F., de Boer, E., Krpic, S., Grosveld, F., and Strouboulis, J. (2009). Optimal use of tandem biotin and V5 tags in ChIP assays. BMC Mol Biol *10*, 6.

Nichols, J., Silva, J., Roode, M., and Smith, A. (2009). Suppression of Erk signalling promotes ground state pluripotency in the mouse embryo. Development (Cambridge, England) *136*, 3215-3222.

Reynolds, N., Salmon-Divon, M., Dvinge, H., Hynes-Allen, A., Balasooriya, G., Leaford, D., Behrens, A., Bertone, P., and Hendrich, B. (2012). NuRD-mediated deacetylation of H3K27 facilitates recruitment of Polycomb Repressive Complex 2 to direct gene repression. The EMBO journal *31*, 593-605.

Wan, H., Luo, F., Wert, S.E., Zhang, L., Xu, Y., Ikegami, M., Maeda, Y., Bell, S.M., and Whitsett, J.A. (2008). Kruppel-like factor 5 is required for perinatal lung morphogenesis and function. Development (Cambridge, England) *135*, 2563-2572.

Wang, W., Lin, C., Lu, D., Ning, Z., Cox, T., Melvin, D., Wang, X., Bradley, A., and Liu, P. (2008). Chromosomal transposition of PiggyBac in mouse embryonic stem cells. Proceedings of the National Academy of Sciences of the United States of America *105*, 9290-9295.

Wray, J., Kalkan, T., Gomez-Lopez, S., Eckardt, D., Cook, A., Kemler, R., and Smith, A. (2011). Inhibition of glycogen synthase kinase-3 alleviates Tcf3 repression of the pluripotency network and increases embryonic stem cell resistance to differentiation. Nat Cell Biol *13*, 838-845.

Wu, T.D., and Nacu, S. (2010). Fast and SNP-tolerant detection of complex variants and splicing in short reads. Bioinformatics *26*, 873-881.
